# Linking peri-implantitis to microbiome changes in affected implants, healthy implants, and saliva: a cross-sectional pilot study

**DOI:** 10.3389/fcimb.2025.1543100

**Published:** 2025-04-17

**Authors:** Lucinda J. Bessa, Conceição Egas, Carolina Pires, Luís Proença, Paulo Mascarenhas, Ricardo J. Pais, Helena Barroso, Vanessa Machado, João Botelho, Gil Alcoforado, José João Mendes, Ricardo Alves

**Affiliations:** ^1^ Egas Moniz Center for Interdisciplinary Research (CiiEM), Egas Moniz School of Health & Science, Almada, Portugal; ^2^ CNC-UC – Center for Neuroscience and Cell Biology, University of Coimbra, UC-Biotech, Cantanhede, Portugal; ^3^ CIBB – Center for Innovative Biomedicine and Biotechnology, University of Coimbra, UC-Biotech, Cantanhede, Portugal; ^4^ Genoinseq – Next Generation Sequencing Unit, Biocant, Cantanhede, Portugal; ^5^ Bioinformatics R&D, Bioenhancer Systems Ltd, Manchester, United Kingdom

**Keywords:** peri-implantitis, peri-implant microbiome, saliva microbiome, functional pathways, shotgun metagenomic sequencing, differential rankings, compositional change

## Abstract

**Introduction:**

The rising use of dental implants is accompanied by an expected increase in peri-implant diseases, particularly peri-implantitis (PI), which poses a significant threat to implant success and necessitates a thorough understanding of its pathogenesis for effective management.

**Methods:**

To gain deeper insights into the role and impact of the peri-implant microbiome in the pathogenesis and progression of PI, we analyzed 100 samples of saliva and subgingival biofilm from 40 participants with healthy implants (HI group) or with co-occurrence of diagnosed PI-affected implants and healthy implants (PI group) using shotgun metagenomic sequencing. We identified the most discriminative species distinguishing healthy from diseased study groups through log ratios and differential ranking analyses.

**Results and discussion:**

*Mogibacterium timidum*, *Schaalia cardiffensis*, *Parvimonas micra*, *Filifactor alocis*, *Porphyromonas endodontalis*, *Porphyromonas gingivalis* and *Olsenella uli* were associated with the subgingival peri-implant biofilm. In contrast, Neisseria sp oral taxon 014, *Haemophilus parainfluenzae*, *Actinomyces naeslundii*, *Rothia mucilaginosa* and *Rothia aeria* were more prevalent in the healthy peri-implant biofilm. Functional pathways such as arginine and polyamine biosynthesis, including putrescine and citrulline biosynthesis, showed stronger correlations with PI-affected implants. In contrast, peri-implant health was characterized by the predominance of pathways involved in purine and pyrimidine deoxyribonucleotide de novo biosynthesis, glucose and glucose-1-phosphate degradation, and tetrapyrrole biosynthesis. Our findings reveal that healthy implants in PI-free oral cavities differ significantly in microbial composition and functional pathways compared to healthy implants co-occurring with PI-affected implants, which more closely resemble PI-associated profiles. This pattern extended to salivary samples, where microbial and functional biomarkers follow similar trends.

## 1 Introduction

Dental implants have become a common and effective solution for treating edentulism, with an annual growth rate of approximately 14%, potentially reaching 23% by 2026 (Kormas et al., 2020). However, as dental implant usage rises, the prevalence of peri-implant diseases is also expected to increase (Wang et al., 2017), which poses a significant threat to the success of dental implants. Thus, it is imperative to clearly define and understand the pathogenesis of these diseases to implement appropriate treatment strategies. Among peri-implant diseases, peri-implantitis (PI) stands out as a major concern and can potentially lead to the loss of dental implants (Berglundh et al., 2024; Schwarz et al., 2021).

The observation that both PI and periodontitis (PD) shares similar inflammatory phenotypes associated with bacterial biofilms and microbial dysbiosis led to the assumption that both diseases have comparable pathogenesis (Meffert, 1996). As a result, treatment approaches for PI are often based on those used for PD. However, this simplistic view was challenged when it became clear that most treatments were ineffective in the long term, as PI frequently recurred after some time (Esposito et al., 2012). This realization, along with insights gained from deep-sequencing studies of periodontal and peri-implant microbiomes (Dabdoub et al., 2013; Komatsu et al., 2020; Yu et al., 2019), led to the establishment that the peri-implant microbial communities are distinct from periodontal ones, imposing a paradigm shift in understanding and treating PI (Kotsakis and Olmedo, 2021; Parga et al., 2024; Zhang et al., 2022).

Due to the complex composition and structure of the oral microbiome, high-throughput sequencing techniques are essential for a comprehensive understanding of the taxonomic and functional profiles of specific niches within it. Although some metagenomic sequencing tools have been employed, most have focused on sequencing the 16S rRNA gene, which primarily provides information about bacterial phylogeny and taxonomy (Barbagallo et al., 2022; Hakkers et al., 2024; Jung and Lee, 2023; Kensara et al., 2023; Kim et al., 2023; Sinjab et al., 2024).

Bacteria play a central role in biofilm formation, acting as primary colonizers and dominating both in abundance and function within a healthy oral microbiome. However, the oral microbiome is a complex ecosystem encompassing fungi, viruses, archaea, and protozoa, all of which must be considered to comprehensively understand the microbial community. Future advancements in this field are likely to hinge on integrative analyses that encompass the complete metagenome and metatranscriptome of the entire microbial consortium.

Komatsu et al. (2020) compared the relative activity/function levels of different species in PI and PD using metagenomic (16S rRNA sequencing) and metatranscriptomic data. To date, there have been limited studies employing shotgun metagenomics to investigate the microbiome in PI (Bazzani et al., 2024; Ghensi et al., 2020; Liang Song et al., 2024; Zhang et al., 2022), and equally few have explored the transcriptome in PI (Becker et al., 2014; Ganesan et al., 2022; Shiba et al., 2016).

Shotgun metagenomic sequencing has more power to identify less abundant taxa and more capacity to identify most microorganisms at the species level than 16S rRNA sequencing, and it allows to obtain the potential functional profiles of the microbial communities analyzed (Durazzi et al., 2021).

In this pilot study, we utilized shotgun metagenomic sequencing to analyze the microbiomes of saliva and subgingival peri-implant biofilms, aiming at identifying distinct microbial signatures and potential functional pathways associated with PI. The study design included patients with healthy implants as well as those with co-occurring PI-affected and healthy implants. Saliva samples were analyzed to evaluate their potential as a non-invasive diagnostic tool for identifying PI biomarkers.

## 2 Materials and methods

### 2.1 Experimental design and ethical aspects

This cross-sectional study is reported based on the 2020 revised PRISMA statement (Page et al., 2021). It was carried out according to the Helsinki Declaration (as per the 2013 revision), and all participants signed a written informed consent form before participation. The study was approved by the Egas Moniz Ethics Committee (process number 1123).

#### 2.1.1 Study population

Participants seeking dental care at the Egas Moniz Dental Clinic (Almada, Portugal) were invited to participate if: aged 18 years old or over; presence of at least one dental implant with a history of at least one year in function; no diagnosis of periodontitis; no history of local or systemic antibiotics or oral antiseptic mouth rinses use within the past 2 months. Women being pregnant or breastfeeding were not included in the study.

The sampling period was from January 2023 to September 2023. Every clinical procedure strictly adhered to the principles outlined in the Declaration of Helsinki and the guidelines of Good Clinical Practice.

#### 2.1.2 Questionnaire

A brief questionnaire was used to gather relevant demographic and clinical information, such as age, sex, pregnancy, presence of immune or inflammatory diseases, regular medication, smoking habits, number of remaining teeth and dental implants, and their location in the oral cavity. All information underwent de-identification before creating the database and conducting data analysis.

### 2.2 Clinical examination

The participants were asked to refrain from oral hygiene for 24 hours and from eating and drinking for 2 hours before the examination and sampling.

To define peri-implant health or PI, the criteria included in the 2018 Classification of Periodontal and Peri-implant Diseases and Conditions (Caton et al., 2018) were used. The diagnosis of a healthy implant involved the following clinical criteria: absence of clinical signs of redness and swelling, absence of bleeding upon probing (except in one location, excluding profuse bleeding), absence of suppuration, absence of increased probing depth (when compared to previous examinations), and absence of radiographic bone loss. After clinical inspection and a final diagnosis, participants were assigned to one of two groups: patients with healthy implants (HI group) or patients with co-occurrence of diagnosed PI-affected implants and healthy implants (PI group).

### 2.3 Sample collection

From each participant, we collected a saliva sample and one or two subgingival peri-implant biofilm samples. In group HI we obtained a saliva sample (HI_Sa) and a subgingival biofilm sample from a healthy implant site (HI_HIS). In group PI, we obtained a saliva sample (PI_Sa), a subgingival biofilm sample from a healthy implant site (PI_HIS), and a subgingival biofilm sample from an implant site affected by PI (PI_PIS). Sampling was performed prior to any antiseptic mouthwash use.

#### 2.3.1 Saliva sampling

Two milliliters of unstimulated saliva were collected from each participant by drooling into a 4-mL cryotube with the aid of a saliva collection aid device (Salimetrics, USA). All samples were immediately transported to the laboratory, where glycerol was added to a final concentration of 20% in aseptic conditions, and then stored at -80°C in aliquots of 1 mL (Marotz et al., 2021).

#### 2.3.2 Subgingival biofilm sampling

After selecting the implant for sampling, the site was isolated using sterile cotton rolls, then the supragingival plaque was removed to avoid cross-contamination with the subgingival biofilm sample that was then collected using sterile PerioPaper (PP) Strips (Oraflow, USA). Each PP strip was gently inserted with a sterile dental forceps into the sulcus or pocket of the implants until a slight resistance was felt. In implants with PI, the site with the greatest probing depth was selected for sample collection. Three PP strips were used per each implant and pooled into 1.5 mL DNase/RNase-free sterile tubes containing 750 µL of sterile solution (50 mM Tris-HCl, pH 7.5; 1 mM EDTA, pH 8.0; 0.5% Tween-20), the same used in the Human Microbiome Project (2010) plus 20% glycerol, and then stored at -80°C for subsequent DNA extraction.

### 2.4 Extraction of total genomic DNA from samples

The extraction of total genomic DNA from frozen saliva and subgingival biofilm samples was performed using the DNeasy PowerSoil Pro kit (Qiagen, Germany). However, a prior step was performed to selectively deplete human DNA, as Marotz et al. (2021) described, with minor modifications and detailed next.

Control extractions were performed using only sample buffer to determine potential contamination during the protocol execution and using the ZymoBIOMICS Microbial Community Standard (Zymo Research Corporation, USA) to validate the efficacy of the extraction method. A total of 100 DNA samples were collected, comprising 40 from saliva and 60 from subgingival biofilm.

#### 2.4.1 Saliva samples

One-mL saliva aliquot was thawed, vortexed, and then centrifuged at 15,000 ×*g*, room temperature, for 6 min to pellet cells. Before DNA extraction, a method was applied to selectively deplete host DNA, based on that described by Marotz et al. (2021). Briefly, the supernatant was removed, the pellet resuspended in 200 μL nuclease-free H_2_O and left at room temperature for 5 min to allow for osmotic lysis of human cells. Propidium monoazide was added to a final concentration of 10 μM, vortexed to mix, and incubated at room temperature, protected from light for 5 min. Then, samples were placed horizontally on ice, at approximately 12 cm from a blue light (wavelength of 480 nm) and exposed for 25 min, briefly vortexing every 5 min. Samples were then centrifuged at 15,000 × *g*, room temperature, for 6 min, and the pellet resuspended in 800 µL of solution CD1 (from the DNeasy PowerSoil Pro kit), immediately transferred to a PowerBead Pro tube of the same kit, briefly vortexed and incubated at 65°C from 10 min and processed according to the manufacturer’s protocol from step 2 on.

#### 2.4.2 Subgingival biofilm samples

Samples were thawed, centrifuged at 15,000 ×*g* for 6 min at 4°C, washed once with 750 µL of PBS 1×, resuspended in 200 μL nuclease-free H_2_O, briefly vortexed and left at room temperature for 5 min. Then, the PMA protocol was the same as described above. Before centrifuging at the end, the PP strips were prior transferred to a PowerBead Pro tube, and the pellet resuspended in 800 µL of solution CD1 was also transferred to the same tube, followed by vortexing and incubation at 65°C from 10 min and then the manufacturer’s protocol from step 2 on.

### 2.5 Library preparation and sequencing

The project consisted of the sequencing of 100 DNA metagenome samples. The concentration of DNA was determined with the Qubit 2.0 Fluorometer (Life Technologies) using the Qubit dsDNA HS Assay Kit (Life Technologies). Each DNA library was prepared from 0.5 nanograms of high-quality genomic DNA with the Nextera XT DNA Sample Preparation Kit (Illumina, San Diego, USA) and paired-end sequenced in the NextSeq 2000 Illumina^®^ sequencer with the NextSeq 1000/2000 P2 XLEAP-SBS Reagent Kit (300 cycles, 2X150 bp) (Illumina, San Diego, CA, USA). All procedures were performed according to standard manufacturer’s protocols.

### 2.6 Read quality control

Sequenced reads were quality-filtered with Trimmomatic version 0.39 (Bolger et al., 2014) using the following parameters: 1) sequencing adapters were removed, 2) bases with an average quality lower than Q25 in a window of 5 bases were trimmed, and 3) reads with less than 100 bases were discarded. High-quality reads were filtered against the reference human genome sequence assembly GRCh38/hg38 with Bowtie version 2.5 (Langmead et al., 2009).

### 2.7 Data analysis and statistics

The demographic and clinical data of patients were analyzed using IBM^®^ SPSS^®^ Statistics v.29, applying comparative statistical tests such as Pearson’s chi-square test or Mann-Whitney, based on variable types and data characteristics. Values of *p* < 0.05 were considered significant.

#### 2.7.1 Taxonomy and diversity analyses

High-quality sequences were analyzed with MetaPhlAn version 4.0.6 (Blanco-Míguez et al., 2023) for determining taxa abundances using the MetaPhlAn clade-specific marker genes mpa_vOct22_CHOCOPhlAnSGB_202212 database. The number of read counts for each taxon identified at the species level per sample was retrieved and used to build an abundance table comprising read counts from all samples. The abundance table was used for composition, alpha and beta diversities and differential abundance analyses.

The number of reads mapping to fungi and viruses on the MetaPhlAn database was very low or even absent. To overcome this limitation, the high-quality reads were analyzed with Kraken2 version 2.1.1 (Wood et al., 2019) against the Viral genomes (2019) and the Fungi genomes (2019) Kraken databases. Abundance estimation at the species level was retrieved with Bracken version 2.9 (Bayesian Reestimation of Abundance with Kraken) (Lu et al., 2017). The number of read counts for each taxon identified per sample was used to build an abundance table comprising information from all samples. The abundance table was used for composition, alpha and beta diversities and differential abundance analyses.

Hill numbers were calculated for Species richness, Shannon and Inverted Simpson with the hilldiv package, version 1.5.1 (Alberdi and Gilbert, 2019). Hill numbers were compared using analysis of variance (ANOVA) followed by Tukey’s test or the Kruskal-Wallis test, followed by Dunn’s test with Bonferroni correction, after testing for normality with the Shapiro test.

Beta diversity was analyzed with Principal Coordinates Analysis (PCoA) in phyloseq using the Bray-Curtis dissimilarity and the Jaccard similarity coefficient. The indexes were tested for statistical differences with PERMANOVA, followed by pairwise PERMANOVA using the adonis function of the vegan package version 2.6-4 (Oksanen et al., 2020) with 1000 permutations and the Benjamini-Hochberg procedure for multiple comparison corrections. Homoscedasticity was tested with the betadisper function of the vegan package.

Beta diversity was further analyzed with the Robust Aitchison distance and Principal Component Analysis (PCA) using DEICODE version 0.2.4 (Martino et al., 2019) within Qiime2 version 2023.5 (Bolyen et al., 2019) from the *biom* abundance files produced by MetaPhlAn, with default parameters. Statistical significance was tested with PERMANOVA and pairwise PERMANOVA within Qiime2 with 999 permutations and the Benjamini-Hochberg procedure for multiple comparison corrections. Dispersion was tested with the permdisp function (also in Qiime2). DEICODE results were visualized with the QIIME2 plugin Emperor (Vázquez-Baeza et al., 2013), identifying the ten most relevant species. Taxa ranks were visualized with Qurro (Fedarko et al., 2020). The 10% most influential species were analyzed according to PCA axis 1 or 2.

The abundance table was additionally analyzed with Songbird version 1.0.4 (Morton et al., 2019) to identify correlations between taxa and study groups. Songbird was run in Qiime2 version 2020.2 in the multinomial mode with the parameters 50,000 epochs, a batch size of 8, a differential prior of 1.0, a minimum sample count of 50 and a minimum feature count of 20% of the samples. Taxa ranks were visualized with QURRO. Log ratios of relevant bacteria were extracted from Qurro. Data visualization and statistical inference analyses were performed using IBM SPSS Statistics v.30.

Alpha diversity (as Hill numbers), beta diversity (Bray-Curtis dissimilarity and Jaccard distance), composition and differential abundance analyses were performed using R Statistical Software version 4.3.0 (R Core Team, 2023) in RStudio version 2023.03.0 build 386 (Posit Team, 2023). Plots were produced with ggplot2 version 3.4.2 (Wickham, 2016). MetaPhlAn and Kraken2 were used in the Galaxy Europe server (The Galaxy Community, 2024).

A p-value and an adjusted p-value of < 0.05 were considered statistically significant.

#### 2.7.2 Functional profiling analyses

High-quality sequences were analyzed with HUMAnN version 3.9 (Beghini et al., 2021) to determine the abundance of the functional pathways present in the metagenomes using the MetaPhlAn clade-specific marker genes mpa_vOct22_CHOCOPhlAnSGB_202212 database. The unstratified functional pathway data was used to build an abundance table comprising read counts from all samples. The abundance table was used for composition and beta diversities analyses (Bray-Curtis dissimilarity, Jaccard similarity coefficient, and Robust Aitchison distance and PCA using DEICODE) and was additionally analyzed with Songbird version 1.0.4 to identify correlations between functional pathways and study groups. All these analyses were performed as described above.

## 3 Results

### 3.1 Clinical and demographic data of the study population

From a total of forty-nine patients that were initially recruited, nine patients were excluded due to insufficient microbial DNA in at least one of their samples, making them unsuitable for further analysis. Consequently, 40 patients with viable samples were included in this study; 20 in the HI group and 20 in the PI group. The demographic and medical characteristics showed no differences between the groups in the analyzed characteristics, except for the total number of dental implants (p=0.005) (**Table 1**).

**Table 1:** Demographic and clinical characteristics of the sampled population (patients in HI and PI groups).

		HI group (n =20)	PI group (n=20)	p-value *
Sex	Female	8 (40.0)	12 (60.0)	0.206 ^a^
	Male	12 (60.0)	8 (40.0)	
Age (years)		64 ± 12	64 ± 9	0.681 ^b^
Smoker	Yes	4 (50.0)	4 (50.0)	1.000 ^a^
	No	16 (50.0)	16 (50.0)	
Former smoker	Yes	5 (50.0)	5 (50.0)	1.000 ^a^
	No	15 (50.0)	15 (50.0)	
Intake of chronic medication	Yes	13 (61.9)	8 (38.1)	0.113 ^a^
	No	7 (36.8)	12 (63.2)	
Chronic and/or coexisting diseases	Yes	14 (56.0)	11 (44.0)	0.465 ^a^
	No	6 (40.0)	9 (60.0)	
Number of remaining teeth		19 ± 8	16 ± 10	0.140 ^b^
Number of dental implants		3 ± 2	6 ± 4	0.005 ^b^

The values are presented as mean ± standard deviation or n (%).*p-value obtained by the tests: ^a^ Pearson’s chi-square test; ^b^ Mann-Whitney test.

### 3.2 Compositional changes and differences in saliva and subgingival peri-implant biofilm samples between the HI and PI groups

#### 3.2.1 Taxonomic composition and diversity features of saliva and peri-implant biofilm microbiomes

From the 100 sequenced samples, a total of 652,464,113 high-quality read pairs were obtained, from which 439,265,781 were non-human read pairs, with an average of 4,392,658 read pairs per sample (ranging from 299,855 to 27,677,702). Sequencing and analysis metrics are shown in **Supplementary Table S1**.

Sequence analysis using MetaPhlAn software identified 596 bacterial species present in at least one sample. In addition, 52 fungal species and 586 viral species (though most viral species were not shared among samples and were present in very low relative abundances) were identified using Kraken2. The vast majority of viruses were bacteriophages.

Overall, regarding the bacterial composition, 13 phyla were identified in the analyzed samples. However, two of them, *Candidatus-Gracilibacteria* and *Deinococcus-Thermus*, were not present in all study groups. The top four most abundant phyla (*Actinobacteria*, *Bacteroidetes*, *Firmicutes*, *Proteobacteria*) embodied over 95% of the total bacterial taxa relative abundances (**Supplementary Figure S1** of **Supplementary Material**). The relative abundances of *Bacteroidetes*, *Candidatus-Saccharibacteria*, and *Fuseobacteria* were higher in the PI-affected implants, PI_PIS group, than in healthy implants HI_HIS and PI_HIS groups (**Supplementary Figure S1** of **Supplementary Material**, **Supplementary Table S2**).

**Figure 1:**
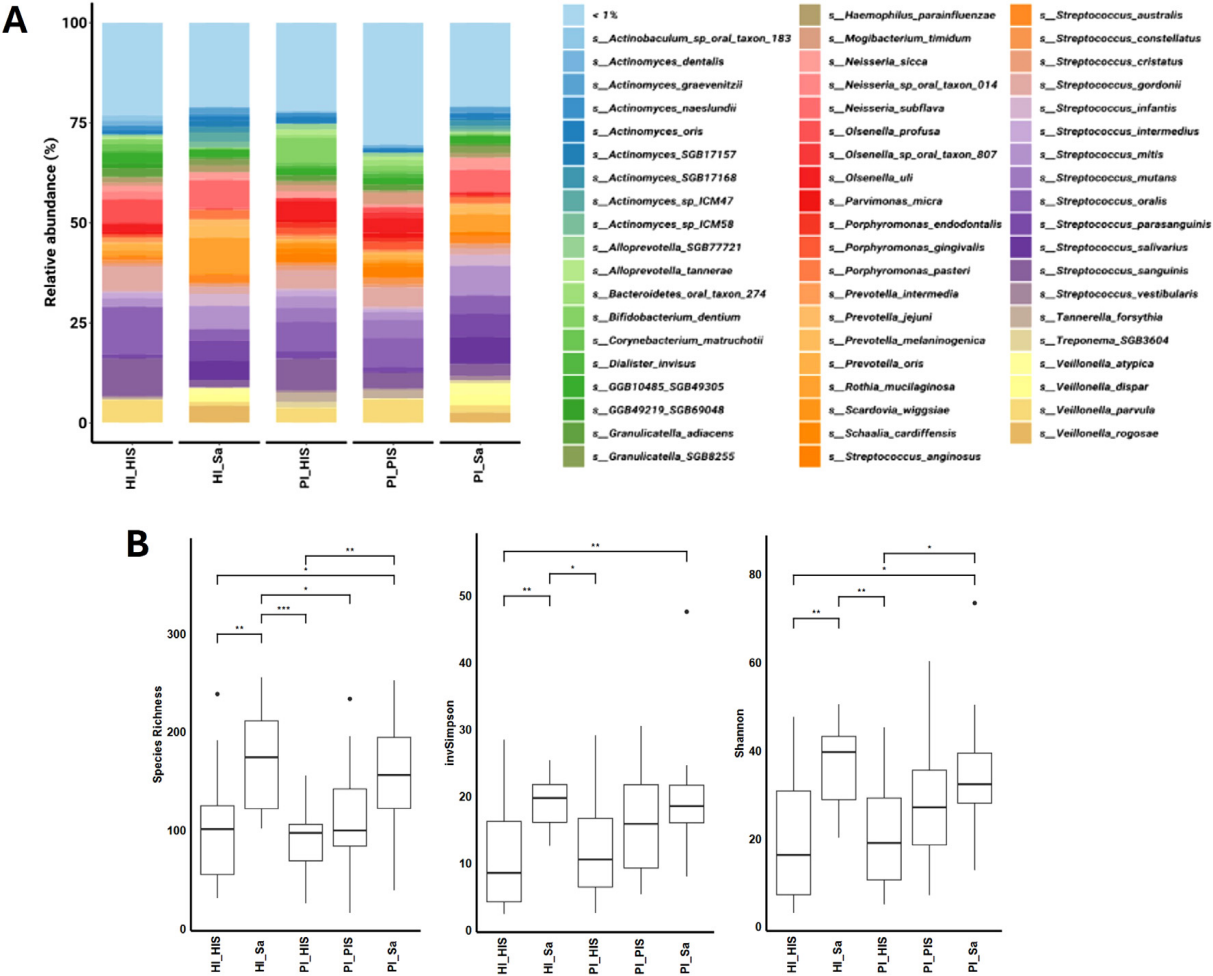
**(A)** Relative abundances (>1%) of the bacterial composition at the species level in each study group. **(B)** Hill diversity indices (Species richness, Shannon and Inverted Simpson) of the bacterial communities of the five study groups. *p < 0.05, **p < 0.01, ***p < 0.001, based on the Kruskal-Wallis rank.

Fifty-eight bacterial species were pinpointed with a relative abundance of > 1% in at least one of the five study groups, as shown in **Figure 1A**. The sum of their average relative abundances was 78.95%, 76.75%, 79.07%, 77.95%, and 69.56% for HI_Sa, HI_HIS, PI_Sa, PI_HIS and PI_PIS groups, respectively. Thus, the PI_PIS group exhibited a greater percentage of species that were present in very low relative abundances compared to the other study groups.

**Supplementary Figures S2A, B** of **Supplementary Material** show the relative abundance (>1%) of all species of fungi and viruses, respectively. *Schizosaccharomyces pombe* was the most abundant fungus in all study groups. Most abundant phages were associated with *Streptococcus* spp.

Hill diversity, which includes species richness and modified versions of the traditional Shannon and Simpson indices, was used to measure the diversity of the bacterial community of each study group (**Figure 1B**). Bacterial Hill-species richness did not differ significantly between the two study groups of saliva nor among the three study groups of subgingival biofilm from implant sites. However, saliva groups had higher species richness (large number of rare species) than subgingival biofilm groups. The Hill-inverted Simpson index, which emphasizes the dominance of common species in a community, was also higher in saliva groups. The Hill-Shannon index balances the influence of species richness and evenness in a community. The Hill-Shannon diversity of the saliva groups (HI_Sa and PI_Sa) was substantially higher than the groups of subgingival biofilms of healthy implants (HI_HIS or PI_HIS). However, the PI_PIS was not statistically different from any other of the four study groups. Thus, the groups showing higher diversity in terms of presenting a higher number of rare species and a higher number of common species were both saliva groups, HI_Sa and PI_Sa, followed by PI_PIS. Overall, comparing the subgingival biofilm groups, they were all homogeneous regarding species richness. However, PI-affected implants tendentially showed bacterial communities with higher Hill-Shannon diversity than healthy implants, although those differences were not statistically significant.

Hill diversity indices were also calculated for fungal and viral communities of each study group (**Supplementary Figures S2C, D** of **Supplementary Material**). However, no statistically significant differences were observed between the two study groups of saliva nor among the three study groups of subgingival biofilm from implant sites. For viruses, as happened for the bacteria, major and significant differences were pinpointed between saliva and subgingival biofilms groups, regardless of the presence of health or disease (PI).

PERMANOVA analysis of beta diversity revealed no significant differences in bacterial community structure based on the Bray-Curtis or the Jaccard indexes between any two of the three groups of subgingival biofilms (HI_HIS *vs* PI_HIS, HI_HIS *vs* PI_PIS, and PI_HIS *vs* PI_PIS) at the species level (**Supplementary Figures S3A, B** of **Supplementary Material**). While Bray-Curtis and Jaccard distance metrics are commonly used to analyze beta diversity in microbiome studies, more recently introduced methods, such as robust Aitchison PCA with DEICODE (Martino et al., 2019), better account for the sparse compositional nature of microbiome datasets, providing enhanced discriminatory power and salient feature ranking between microbial niches. Therefore, beta diversity was also assessed using DEICODE, however, no statistical significance was obtained, after calculating PERMANOVA (**Supplementary Table S1** of **Supplementary Material**) between the following groups (HI_Sa *vs* PI_Sa, HI_HIS *vs* PI_HIS, HI_HIS *vs* PI_PIS, and PI_HIS *vs* PI_PIS) (**Supplementary Figure S4** of **Supplementary Material**).

#### 3.2.2 Differential ranking and log ratios reveal differences in bacterial species abundance between two study groups

**Figure 2:**
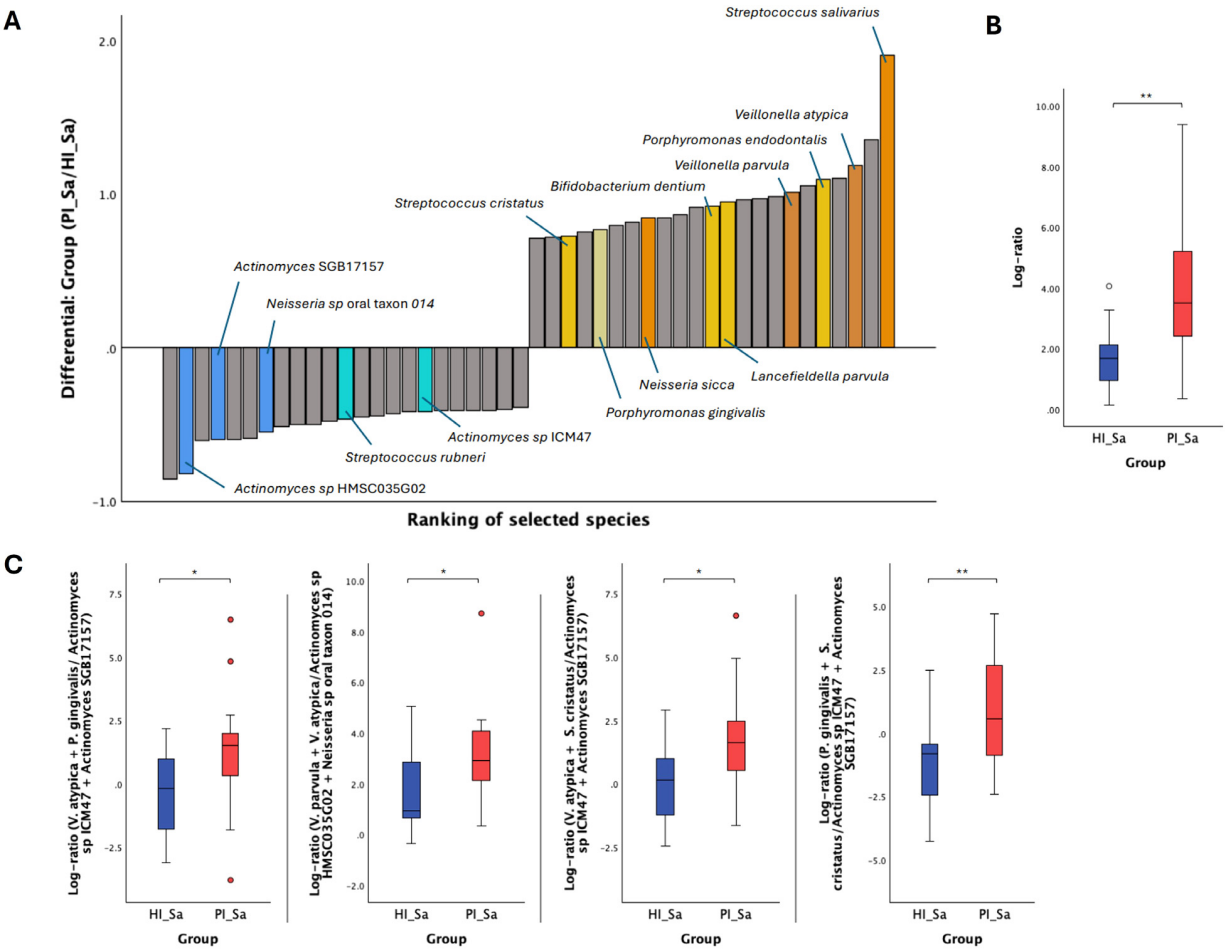
Significant taxonomic differences in the oral metagenome of saliva from healthy and PI-affected patients (HI_Sa *vs* PI_Sa). **(A)** Bacterial differential ranks of the 23 out of 231 (9.96%) species more (identified as numerator) and less (identified as denominator) associated with PI_Sa using the group HI_Sa as reference, as estimated from multinomial regression by Songbird. **(B)** Log ratio plots of the 23 out of 231 species across HI_Sa and PI_Sa groups. **(C)** Log ratio plots of specific combinations of bacterial species across HI_Sa and PI_Sa groups. Statistical significance based on a Student’s t-test (*p < 0.05; **p < 0.01).

Due to the compositional nature of sequencing data, using log ratios can be a more effective method for analyzing differences within these data sets (Morton et al., 2019). Therefore, Songbird was used to perform multinomial regression (Morton et al., 2019) to obtain differential rankings of taxa that are changing the most between the two study groups being compared. In **Figure 2A** and **Supplementary Table S3**, we can observe the differential ranking of the 10% of all species (23 out of 231) that are changing the most between HI_Sa and PI_Sa, with 23 species that go into the “top” (numerator) and other 23 into the “bottom” (denominator) part of the log ratio calculation. In addition, the log ratio of those selected species was significantly increased in PI_Sa than in HI_Sa (t-test, p < 0.01), as shown in **Figure 2B**. Therefore, the species identified as numerator were more associated with PI_Sa and less with HI_Sa. The contrary happens for the species identified as denominator. Moreover, some species were more or less enriched across the samples of each group if they present a positive or negative intercept, respectively (**Supplementary Table S3**). Based on that, and apart from several species of *Streptococcus* (*S. salivarius*, *S. anginosus*, *S. vestibularis*, *S. mitis* and *S. parasanguinis*), we highlighted, as indicated in **Figure 2A**, other species more associated with the PI_Sa, such as *Veillonella atypica* and *Veillonella parvula*, *Porphyromonas endodontalis*, *Porphyromonas gingivalis*, *Bifidobacterium dentium*, and *Neisseria sicca*. Instead, *Actinomyces* spp. were more associated with HI_Sa. Moreover, the log ratios of *Veillonella parvula* + *Veillonella atypica* to *Actinomyces* sp HMSC035G02 + *Neisseria* sp oral taxon 014, *Veillonella atypica* + *Porphyromonas gingivalis* to *Actinomyces* sp ICM47 + *Actinomyces* SGB17157, and *Porphyromonas gingivalis* + *Streptococcus cristatus* to *Actinomyces* sp ICM47 + *Actinomyces* SGB17157 were significantly higher in PI_Sa in comparison to HI_Sa (**Figure 2C**).

**Figure 3:**
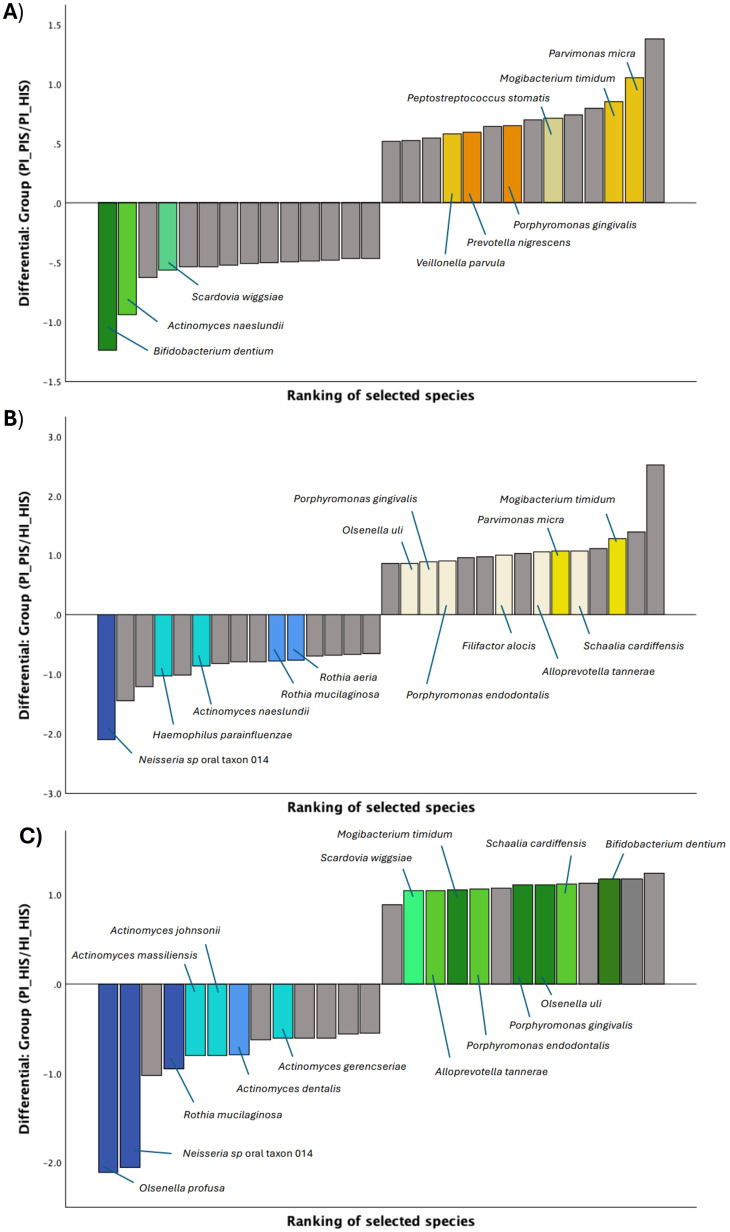
Differential ranks of the 10% species changing the most relative to each other in the two compared groups of subgingival biofilms, as estimated from multinomial regression by Songbird. **(A)** 14 out of 145 species presenting very different ranks in PI_HIS *vs* PI_PIS. **(B)** 15 out of 159 species presenting very different ranks in HI_HIS *vs* PI_PIS. **(C)** 13 out of 137 species presenting very different ranks in HI_HIS *vs* PI_HIS.

Similarly, log ratios were calculated for the subgingival biofilm groups. **Figure 3** displays the differential rankings of the top and bottom 10% of species that changed the most relative to each other between the two groups being compared (see also **Supplementary Tables S4**-**S6**). Key species were highlighted based on their ranking and/or enrichment (positive and higher intercept).

As shown in **Figure 3A**, *Parvimonas micra*, *Mogibacterium timidum*, *Veillonella parvula*, *Porphyromonas gingivalis* and *Peptostreptococcus stomatis* were identified as numerators, thus more associated with PI_PIS, and presented higher ranks in contrast to *Bifidobacterium dentium*, *Actinomyces naeslundii* and *Scardovia wiggsiae* which had low ranks and were identified as denominators, thus more associated with PI_HIS. In turn, some of the species more associated with PI_PIS, using the group HI_HIS as reference, were *Mogibacterium timidum*, *Schaalia cardiffensis*, *Parvimonas micra*, *Filifactor alocis*, *Porphyromonas endodontalis*, *Porphyromonas gingivalis* and *Olsenella uli*, and the species less associated with PI_PIS and, thus, associated with HI_HIS were *Neisseria* sp oral taxon 014, *Haemophilus parainfluenzae*, *Actinomyces naeslundii*, *Rothia mucilaginosa* and *Rothia aeria* (**Figure 3B**). From the differential ranking of the species associated with PI_HIS using group HI_HIS as reference, we found *Bifidobacterium dentium*, *Schaalia cardiffensis*, *Olsenella uli*, *Porphyromonas gingivalis* and *Mogibacterium timidum*, among others, as highlighted in **Figure 3C**. While *Olsenella profusa*, *Neisseria* sp oral taxon 014*, Rothia mucilaginosa*, and several species of *Actimomyces* were more associated with HI_HIS.

**Figure 4:**
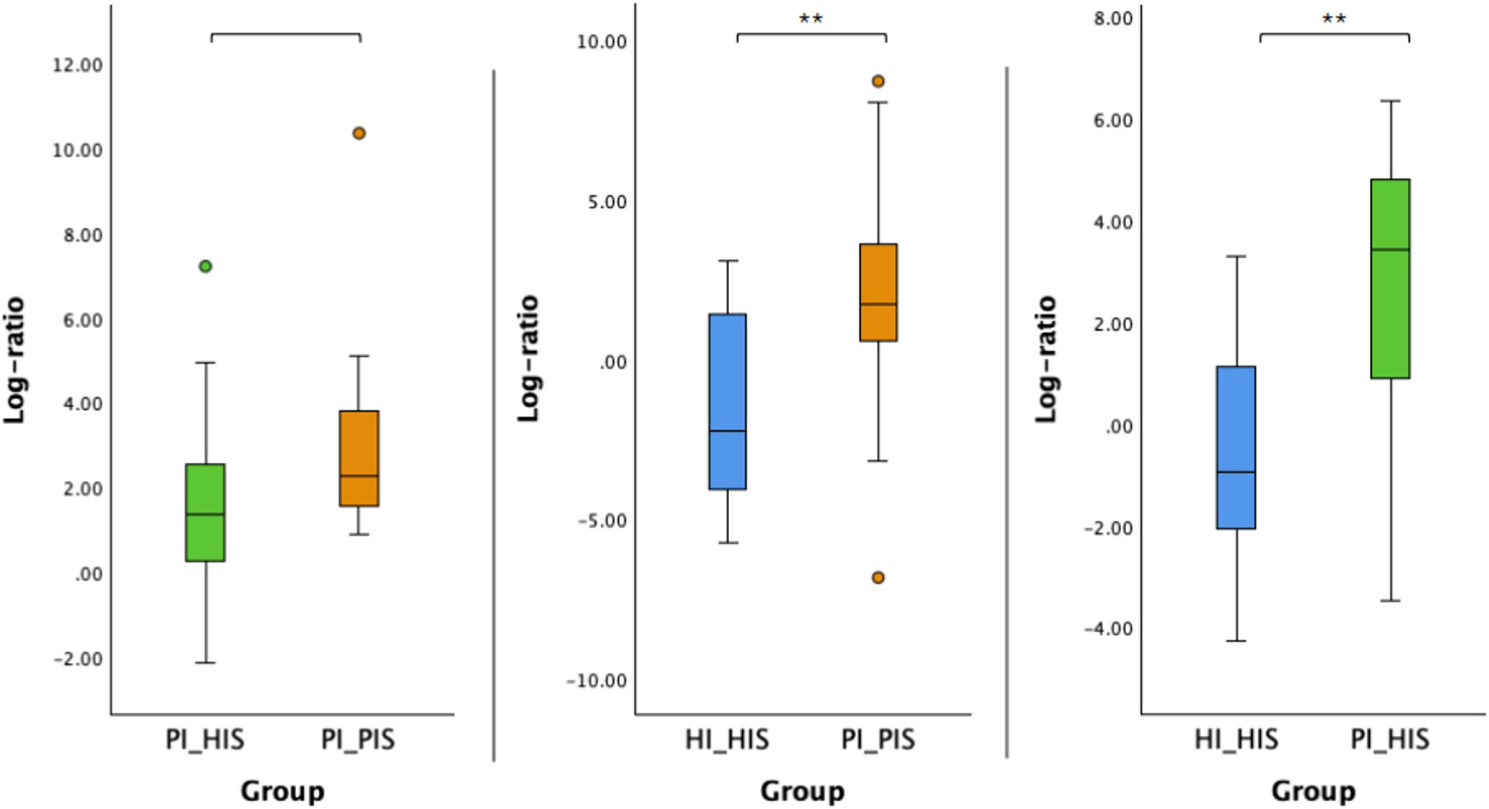
Log ratios of the top and bottom 10% species changing the most across the two groups in comparison. Statistical significance based on a Student’s t-test (**p < 0.01).

The log ratio of all species selected at the 10% threshold was significantly higher in PI_PIS compared to HI_HIS and in PI_HIS compared to HI_HIS (p < 0.01). Although an increase was also observed in PI_PIS compared to PI_HIS, this difference did not reach statistical significance (p = 0.054), as shown in **Figure 4**.

**Figure 5:**
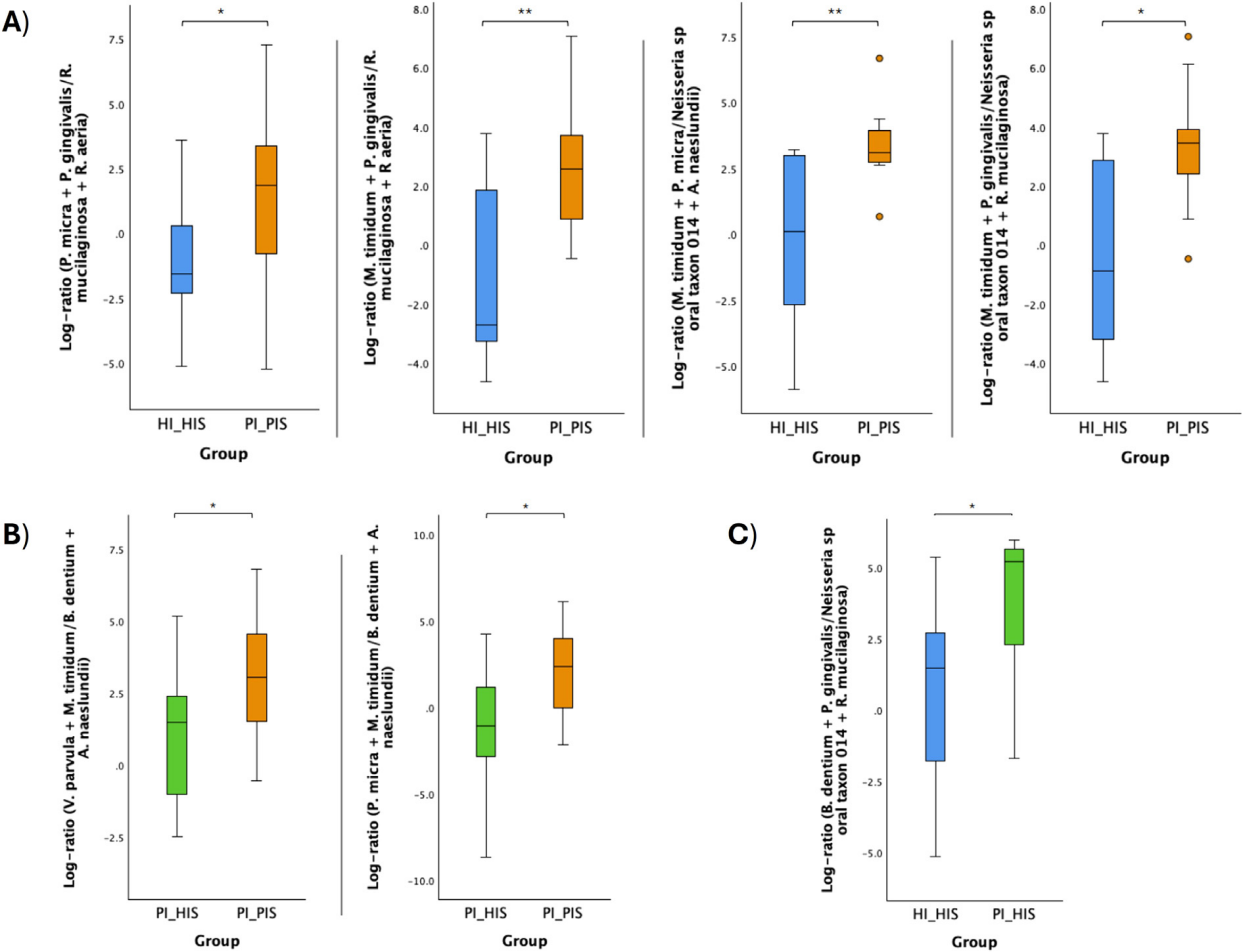
Log ratio plots of specific combinations of bacterial species across HI_HIS and PI_PIS groups **(A)**, PI_HIS and PI_PIS groups **(B)**, and HI_HIS and PI_HIS **(C)**. Statistical significance based on a Student’s t-test (*p < 0.05; **p < 0.01).

Log ratios of specific bacterial species combinations were found to differ significantly between the groups PI_HIS and PI_PIS, HI_HIS and PI_PIS, and HI_HIS and PI_HIS (**Figure 5**). Log ratios of: *Mogibacterium timidum* + *Porphyromonas gingivalis* to *Neisseria* sp oral taxon 014 + *Rothia mucilaginosa*; *Parvimonas micra* + *Porphyromonas gingivalis* to *Rothia mucilaginosa* + *Rothia aeria*; *Mogibacterium timidum* + *Porphyromonas gingivalis* to *Rothia mucilaginosa* + *Rothia aeria*; and *Mogibacterium timidum* + *Parvimonas micra* to *Neisseria* sp oral taxon 014 + *Actinomyces naeslundii* were all significantly high in the subgingival biofilms of PI-affected implants (PI_PIS) than in healthy implants from patients without any implant with the diagnostic of the disease (HI_HIS) (**Figure 5A**).The log ratios of *Veillonella parvula* + *Mogibacterium timidum* to *Bifidobacterium dentium* + *Actinomyces naeslundii*, and *Parvimonas micra* + *Mogibacterium timidum* to *Bifidobacterium dentium* + *Actinomyces naeslundii* were significantly high in the subgingival biofilms of PI-affected implants than in healthy implants co-occurring within the same patient (**Figure 5B**). Only the log ratio of *Bifidobacterium dentium* + *Porphyromonas gingivalis* to *Neisseria* sp oral taxon 014 + *Rothia mucilaginosa* was found to be significantly increased in PI_HIS in comparison to HI_HIS (**Figure 5C**), which reinforces the differences in the microbiome of healthy implants depending on whether there is co-presence of PI-affected implants in the same oral cavity or not.

#### 3.2.3 Differential ranking and log ratios did not highlight differences in fungal or viral species abundance between two study groups

Differential ranks for the top 10% most and least influential fungal species associated with the disease group, using the healthy group as a reference, were also obtained (see **Supplementary Table S7**). These species, identified as numerator or denominator, were used to calculate log ratios; however, no statistically significant differences were observed in the log ratios between the two groups compared, whether in saliva or subgingival biofilm. Nonetheless, *Candida albicans* was consistently identified as the numerator associated with disease groups PI_Sa (compared to HI_Sa) and PI_PIS (compared to either PI_HIS or HI_HIS).

Similarly, regarding the viruses, differential ranks were obtained, and we could identify which species were changing the most relative to each other, however, those species were mostly bacteriophages and the log ratios calculated were not statistically different across compared groups (data not shown).

### 3.3 Functional profiling changes in saliva and subgingival peri-implant biofilm samples between the HI and PI groups

In total, 406 functional pathways were identified by HUMAnN across all samples and the abundance of those functional pathways present in the metagenomes was also provided (see **Supplementary Table S8**). No significant differences were observed in the beta diversity (analyzed by using Bray-Curtis dissimilarity and the Jaccard similarity coefficients and DEICODE) of the functional pathways when comparing HI_Sa *vs* PI_Sa, HI_HIS *vs* PI_PIS, HI_HIS *vs* PI_HIS and PI_HIS *vs* PI_PIS (**Supplementary Figure S5** of **Supplementary Material**).

Then, the functional pathways present in the oral microbiomes of each study group were examined through differential ranks and log ratio calculation through multinomial regression in Songbird. Differential ranking of the top and bottom 10% of functional pathways, categorized as more associated (numerator) and less associated (denominator), respectively, with one disease associated-study group, using a healthy associated-study group as a reference were obtained and are presented in **Supplementary Tables S9**-**S12**. Out of the analyzed functional pathways, 30 of 302 were selected for the HI_Sa and PI_Sa groups, 29 of 297 for PI_HIS and PI_PIS, 29 of 292 for HI_HIS and PI_PIS, and 28 of 289 for HI_HIS and PI_HIS.

Several functional pathways were consistently more associated with the PI condition, including the biosynthesis of arginine and polyamine, L-citrulline, putrescine, biotin and fructan, as well as the degradation of purine nucleobases. Other pathways could be pinpointed as health-promoting, including the *de novo* biosynthesis of purine and pyrimidine deoxyribonucleotides, biosynthesis of heme b, biosynthesis of tetrapyrrole, and biosynthesis of sulphur amino acids (cysteine and L-methionine).

**Figure 6:**
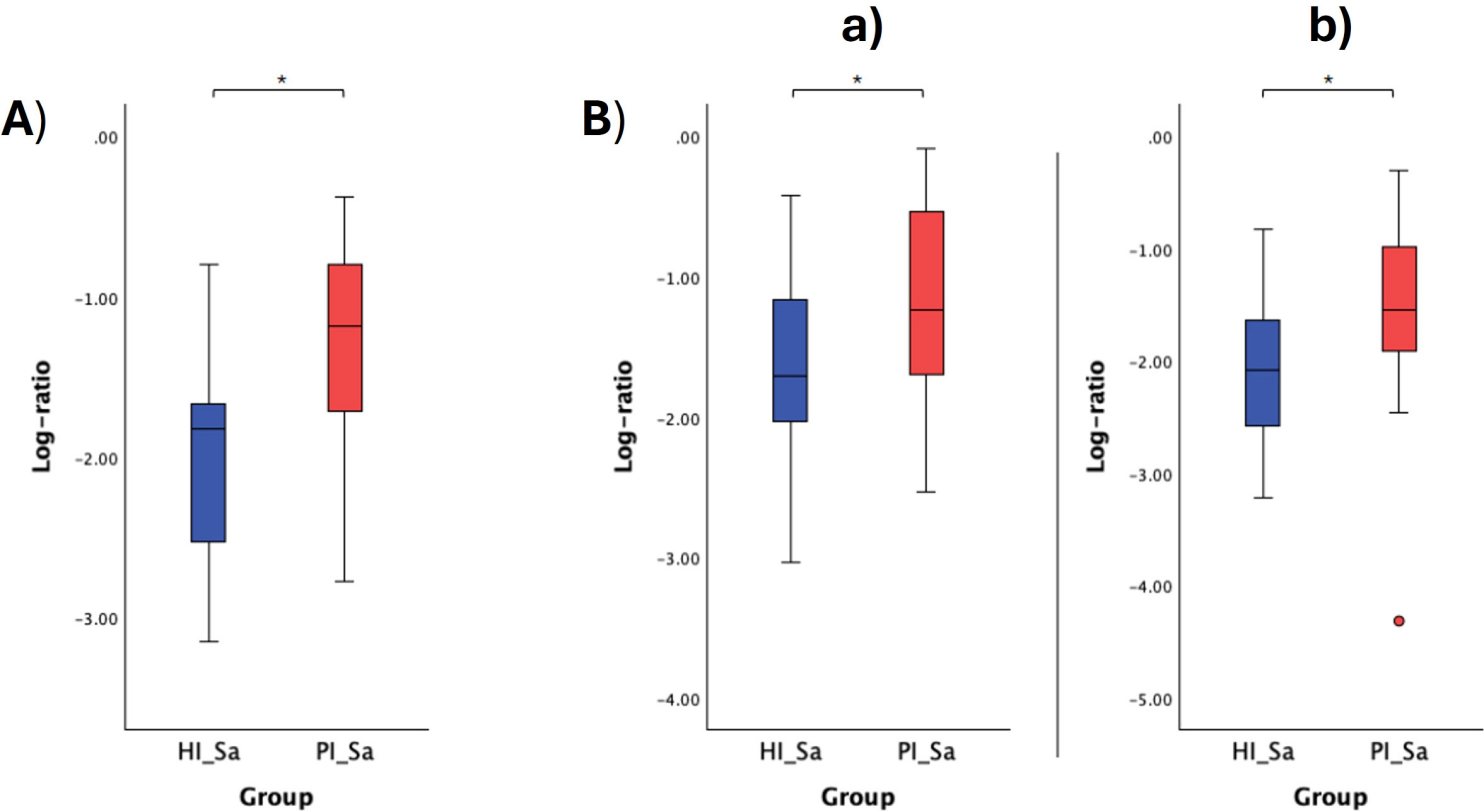
Log ratio plots of the top and bottom 10% functional pathways selected across HI_Sa and PI_Sa **(A)**, and log ratio plots of specific combinations of functional pathways across HI_Sa and PI_Sa **(B)**. [a): log (ARG+POLYAMINE-SYN: superpathway of arginine and polyamine biosynthesis + PWY-5005: biotin biosynthesis II + PWY-822: fructan biosynthesis/PWY-1269: CMP-3-deoxy-D-manno-octulosonate biosynthesis + PANTO-PWY: phosphopantothenate biosynthesis I + PWY-5840: superpathway of menaquinol-7 biosynthesis); b): log (PWY-6305: superpathway of putrescine biosynthesis + PWY-7254: TCA cycle VII (acetate-producers) + PWY-822: fructan biosynthesis/PWY-1269: CMP-3-deoxy-D-manno-octulosonate biosynthesis + PANTO-PWY: phosphopantothenate biosynthesis I + PWY-5840: superpathway of menaquinol-7 biosynthesis)]. Statistical significance based on a Student’s t-test (*p < 0.05).

**Figure 7:**
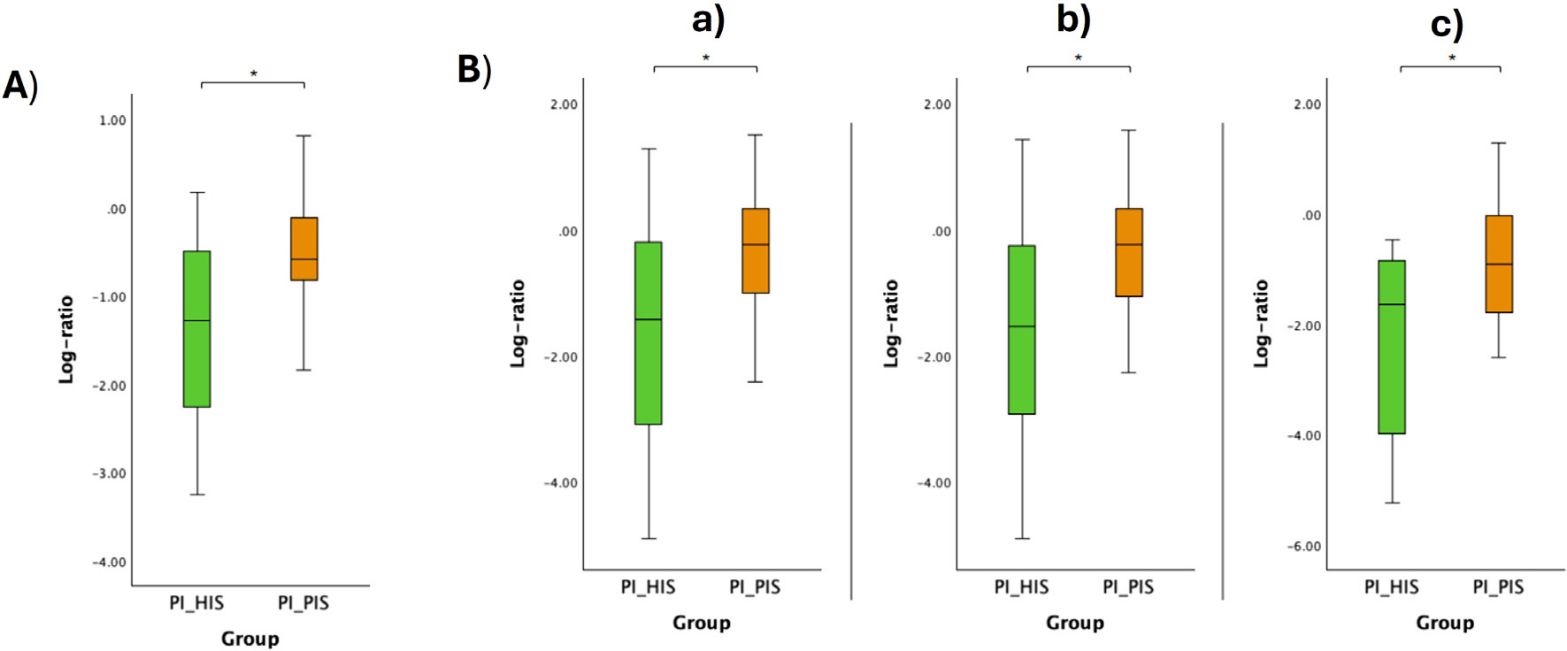
Log ratio plots of the top and bottom 10% functional pathways selected across PI_HIS and PI_PIS **(A)**, and log ratio plots of specific combinations of functional pathways across PI_HIS and PI_PIS **(B)**. [a): log (BIOTIN-BIOSYNTHESIS-PWY: biotin biosynthesis I + CITRULBIO-PWY: L-citrulline biosynthesis + ARG+POLYAMINE-SYN: superpathway of arginine and polyamine biosynthesis/P124-PWY: Bifidobacterium shunt + PWY-7013: (S)-propane-1,2-diol degradation + PWY-7210: pyrimidine deoxyribonucleotides biosynthesis from CTP); b): log (BIOTIN-BIOSYNTHESIS-PWY: biotin biosynthesis I + PWY-6305: superpathway of putrescine biosynthesis + CITRULBIO-PWY: L-citrulline biosynthesis/P124-PWY: Bifidobacterium shunt + PWY-7013: (S)-propane-1,2-diol degradation + PWY-7210: pyrimidine deoxyribonucleotides biosynthesis from CTP); c): log (ARG+POLYAMINE-SYN: superpathway of arginine and polyamine biosynthesis + PWY-6305: superpathway of putrescine biosynthesis + CITRULBIO-PWY: L-citrulline biosynthesis/P124-PWY: Bifidobacterium shunt + P185-PWY: formaldehyde assimilation III (dihydroxyacetone cycle) + PWY-7210: pyrimidine deoxyribonucleotides biosynthesis from CTP]. Statistical significance based on a Student’s t-test (*p < 0.05).

**Figure 8:**
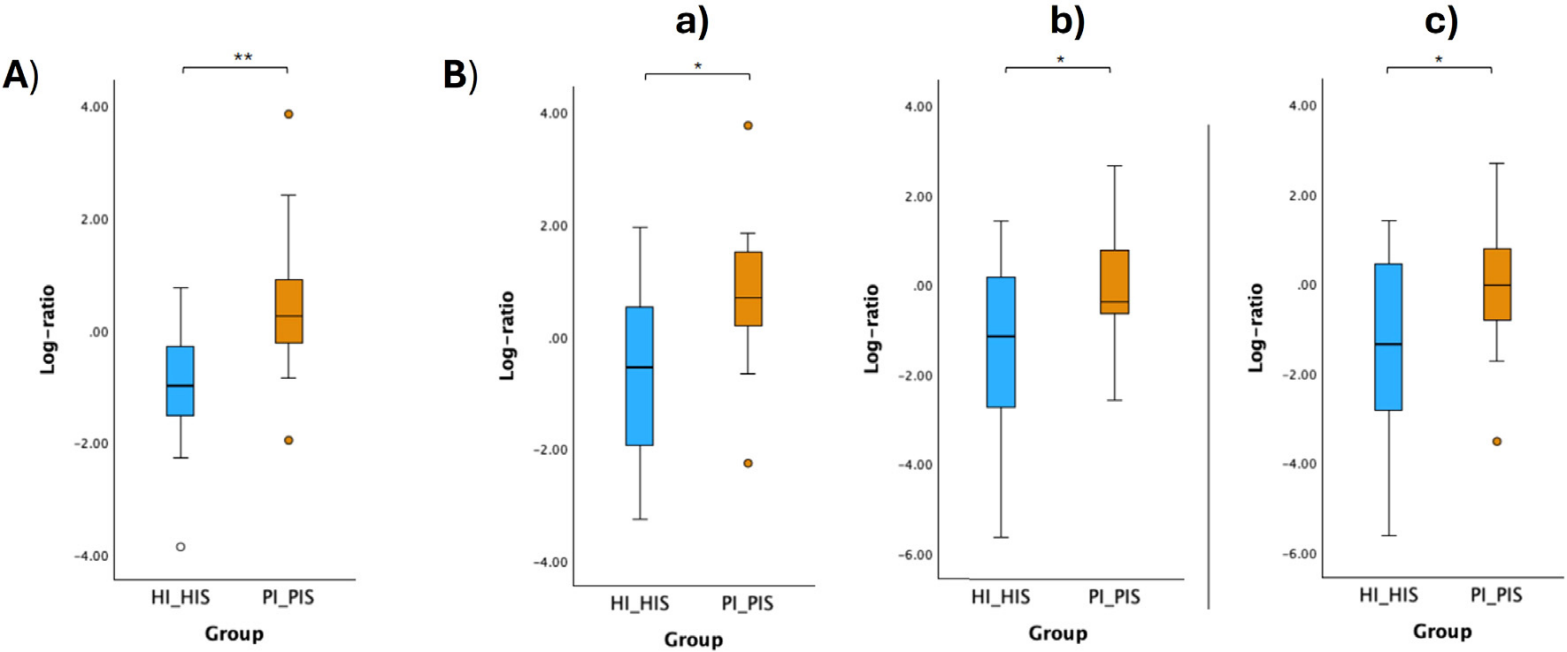
Log ratio plots of the top and bottom 10% functional pathways selected across HI_HIS and PI_PIS **(A)**, and log ratio plots of specific combinations of functional pathways across HI_HIS and PI_PIS **(B)**. [a): log (ARGININE-SYN4-PWY: L-ornithine biosynthesis II + CITRULBIO-PWY: L-citrulline biosynthesis + PWY0-1297: superpathway of purine deoxyribonucleosides degradation/GLUCOSE1PMETAB-PWY: glucose and glucose-1-phosphate degradation + PWY-7013: (S)-propane-1,2-diol degradation + PWY-7883: anhydromuropeptides recycling II); b): log (P164-PWY: purine nucleobases degradation I (anaerobic) + ARG+POLYAMINE-SYN: superpathway of arginine and polyamine biosynthesis + PWY-5838: superpathway of menaquinol-8 biosynthesis I/PWY-5189: tetrapyrrole biosynthesis II (from glycine) + GLUCOSE1PMETAB-PWY: glucose and glucose-1-phosphate degradation + DENOVOPURINE2-PWY: superpathway of purine nucleotides *de novo* biosynthesis II); c): log (P164-PWY: purine nucleobases degradation I (anaerobic) + ARG+POLYAMINE-SYN: superpathway of arginine and polyamine biosynthesis + CITRULBIO-PWY: L-citrulline biosynthesis/PWY-5189: tetrapyrrole biosynthesis II (from glycine) + GLUCOSE1PMETAB-PWY: glucose and glucose-1-phosphate degradation + DENOVOPURINE2-PWY: superpathway of purine nucleotides *de novo* biosynthesis II]. Statistical significance based on a Student’s t-test (*p < 0.05; **p < 0.01).

**Figure 9:**
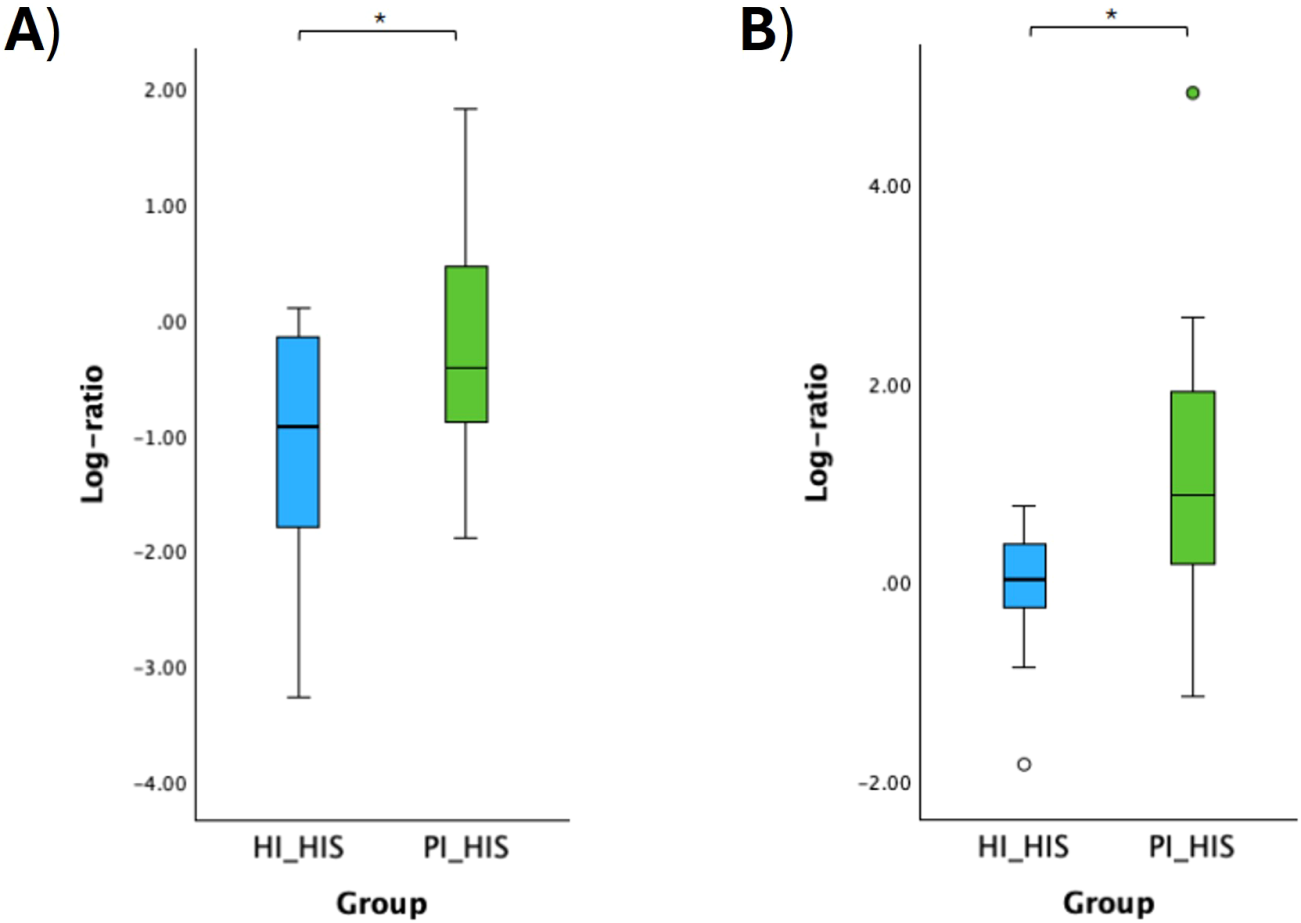
Log ratio plots of the top and bottom 10% functional pathways selected across HI_HIS and PI_HIS **(A)**. Log ratio plots of a specific combination of functional pathways across HI_HIS and PI_HIS **(B)**: log [PWY-5838: superpathway of menaquinol-8 biosynthesis I + PWY0-1297: superpathway of purine deoxyribonucleosides degradation + P124-PWY: Bifidobacterium shunt/PWY-5918: superpathway of heme b biosynthesis from glutamate + PWY-8131: 5’-deoxyadenosine degradation II + PWY-5189: tetrapyrrole biosynthesis II (from glycine)]. Statistical significance based on a Student’s t-test (*p < 0.05).

The respective log ratios were also calculated and significant differences were observed between the two groups compared (**Figures 6A**, **7A**, **8A**, **Figure 9A**). Notably, the significant differences observed between the healthy implants in both groups (HI_HIS and PI_HIS) underscore distinct functional profiles depending on whether PI-affected implants are also present in the same oral cavity. Combinations of two pathways in the numerator and denominator did not yield statistically significant differences. However, log ratios involving combinations of three pathways showed significant differences between the groups HI_Sa and PI_Sa (**Figure 6B**), PI_HIS and PI_PIS (**Figure 7B**), HI_HIS and PI_PIS (**Figure 8B**). Only the log ratio of one combination differed significantly between groups HI_HIS and PI_HIS (**Figure 9B**).

## 4 Discussion

### 4.1 No distinct microbial diversity between healthy and diseased communities

The analysis of the abundances of the bacteria, fungi and viruses present in the PI- and health-associated microbiomes revealed no significant differences in alpha and beta diversity at the species level, although PI-affected implants tendentially showed bacterial communities with higher Hill-Shannon diversity. Previous studies have demonstrated that clinically confirmed PI is associated with greater microbial diversity compared to healthy peri-implant sites (Sanz-Martin et al., 2017; Kröger et al., 2018; Song et al., 2022; Korsch et al., 2021; Di Spirito et al., 2024). However, other studies reported a less diverse microbiome in PI with fewer dominant species compared to healthy implants (Ghensi et al., 2020; Kensara et al., 2023; Liang Song et al., 2024). These disagreements between studies can be explained by the variations in DNA extraction and sequencing methodologies, bioinformatic and/or data analysis approaches. The use of standardized or widely accepted bioinformatic tools and parameters for data processing and analysis should be prioritized. Nonetheless, metagenomic and bioinformatic techniques are evolving rapidly, necessitating continuous adaptation to incorporate the latest methodologies. Microbiome sequencing data, including 16S rRNA and whole metagenome data, are inherently compositional, making differential abundance analysis challenging. Given this compositional nature, log ratios are being reported as a preferred method for examining differences within these datasets, significantly enhancing the accuracy and reliability of inference (Sun et al., 2023; Morton et al., 2019; Mandal et al., 2015).

### 4.2 Distinct bacterial species combinations associated with healthy and PI communities

Differential rank and log ratio analyses of saliva samples identified several species more prevalent in saliva associated with PI compared to the healthy condition, including *Veillonella parvula*, *Veillonella atypica*, *Porphyromonas gingivalis*, and *Streptococcus cristatus*. In contrast, several *Actinomyces* species, such as *Actinomyces* sp HMSC035G02 and *Actinomyces* sp ICM47, and *Neisseria* sp oral taxon 014 were more closely associated with saliva from individuals without PI (HI group). *Veillonella* spp. have frequently been associated with healthy implant sites (Giok and Menon, 2023; Kensara et al., 2023; Sanz-Martin et al., 2017; Sousa et al., 2017). However, a study by Kim et al. (2023) reported higher levels of *Veillonella* in the subgingival biofilm of PI compared to healthy and PD subgingival biofilms. Similarly, another study also linked an increase in *Veillonella* abundance to PI (Daubert et al., 2018). Moreover, *Veillonella atypica* has also been shown to produce heme, serving as a preferred iron source for *Porphyromonas gingivalis* (Zhou et al., 2016). More recently, *Veillonella* spp. were proposed to behave as “accessory pathogens” (Zhou et al., 2021). Interestingly, our results highlighted a higher log ratio of *Veillonella atypica* + *Porphyromonas gingivalis* to *Actinomyces* sp. ICM47 + *Actinomyces* SGB17157 in the saliva of the PI group compared to the HI group. This suggests that these species, detectable in saliva, may serve as potential biomarkers for PI.

Likewise, the log ratio of *Porphyromonas gingivalis* + *Streptococcus cristatus* to *Actimomyces* species was also higher in the saliva of PI group. An antagonistic relationship between *Streptococcus cristatus* and *Porphyromonas gingivalis* through cell-cell communication has been previously demonstrated *in vitro* (Ho et al., 2017). The same research group recently reported that dental plaques from PD patients with low *S. cristatus*/*P. gingivalis* ratios exhibited elevated levels of several well-established periodontitis-associated bacteria, reduced levels of *Streptococcus* spp. and *Actinomyces* spp., and a diverse microbial composition with enhanced antibiotic resistance gene profiles (Wang et al., 2024). These findings position *S. cristatus* and *P. gingivalis* as core bacterial species in the dental plaque microbiome. However, their specific roles in the PI microbiome remain unclear. Our results, showing elevated levels of a combination of *P. gingivalis* and *S. cristatus* relative to *Actinomyces* spp. in the saliva of PI patients, provide a foundation for further investigation into their potential contributions to PI pathogenesis.

We identified bacterial species more strongly associated with the subgingival microbiome of implants affected by PI (PI_PIS) compared to healthy implants in PI-free oral cavities (HI_HIS). For instance, bacteria like *Mogibacterium timidum*, *Parvimonas micra*, *Porphyromonas gingivalis*, *Olsenella uli*, *Porphyromonas endondotalis* and *Filifactor alocis* were ranked higher in PI_PIS relative to other species. Whereas *Rothia aeria*, *Rothia mucilaginosa*, *Actinomyces naeslundii*, *Haemophilus parainfluenzae* and *Neisseria* sp oral taxon 014 were some of the species with higher differential ranks in HI_HIS. *Porphyromonas* spp. have been reported at PI sites in multiple studies utilizing a variety of methodologies (Bazzani et al., 2024; Ghensi et al., 2020; Kim et al., 2023; Liang Song et al., 2024). *Filifactor alocis*, and *Parvimonas micra* have been consistently reported to exhibit increased abundance and prevalence in PI microbiomes (Sanz-Martin et al., 2017; Kim et al., 2023; Song et al., 2024). In addition, *Mogibacterium* spp. have also been linked to PI, although their association has been documented in a smaller number of studies (Kim et al., 2023; Sousa et al., 2017). *Olsenella uli* has also been associated with PI in the study of Kim et al. (2023). The species we identified as associated with a healthy implant align with findings from numerous studies, which also report the presence of *Neisseria* spp., *Rothia* spp., *Actinomyces* spp., and *Haemophilus* spp. at healthy implant sites (Gazil et al., 2022; Giok and Menon, 2023; Song et al., 2024).

Few studies have used intra-subject healthy and PI-affected implants. Our findings suggest that the microbiomes of healthy implants and PI-affected implants in patients with co-occurrence of both conditions are not as distinct from each other as the microbiomes of healthy implants in patients without any PI-affected implants compared to PI-affected implants. Moreover, our results showed that the microbiome of healthy implants differed significantly depending on the presence or absence of PI-affected implants within the same oral cavity. In the study of Ghensi and collaborators (Ghensi et al., 2020), which analyzed the plaque microbiome associated with PI and mucositis in a cohort of 72 patients using metagenomic sequencing, the authors included various controls. Those controls consisted of healthy implants and teeth sampled from healthy sites in healthy individuals, and contralateral healthy sites relative to the mucositis or PI sites. They concluded that the PI microbiome was site-specific, as contralateral healthy sites more closely resembled the microbiome of healthy implants. One potential explanation for the differing findings between the study by Ghensi et al. (2020) and our study could be the distinct bioinformatics analyses conducted in both studies. Ghensi et al. (2020) used linear discriminant analysis (LDA) effect size (LEfSe) method to identify biomarkers that were significantly different between two or more groups, while we have used log ratios and differential ranking with Songbird as described by Morton et al. (2019). A recent systematic review concluded that sequencing-based studies analyzing the peri-implant microbiome have yet to establish a clear and distinct microbial profile, and additional studies with greater standardization are required to enable meaningful comparisons of findings (Giok and Menon, 2023).

### 4.3 Different functional pathways linked to healthy and PI communities

Functional pathways capture the collective metabolic activities of microbial communities. Therefore, changes in the microbiome are reflected in changes in functional pathways, as shown by our results. We could find combinations of functional pathways that were more linked to PI and others to peri-implant health. Pathways associated with arginine and polyamine biosynthesis, putrescine, and citrulline biosynthesis were correlated with PI.

Arginine serves as a precursor for both citrulline (via nitric oxide metabolism) and putrescine (via polyamine biosynthesis), so these molecules are linked through regulated metabolic pathways, whose enhanced activity may reflect microbial adaptations or host responses in conditions like PI. Polyamines have been shown to contribute to bacterial pathogenicity and the formation of biofilms (Banerji et al., 2021). The pathway of biotin biosynthesis, fructan biosynthesis, and the superpathway of purine deoxyribonucleosides degradation were also correlated to PI. A microbiome with microbes capable of biotin synthesis may contribute to inflammation (Yang et al., 2023). Biotin functions not only as a coenzyme in metabolic processes but also modulates intracellular signaling pathways and regulates the expression of metabolic enzymes. While biotin has been linked to various inflammatory diseases, the precise mechanisms behind these associations are not yet fully understood (Sakurai-Yageta and Suzuki, 2024). Regarding fructan, it is known that microbial fructan, comprising about 30% of the extracellular polymeric substances matrix in dental plaque biofilms, protects cells from antimicrobial agents and immune response (Ko et al., 2022).

Peri-implant health was characterized by metabolic pathways that support microbial growth and homeostasis, and nutrient metabolism. For instance, carbohydrate metabolism is essential for energy production, supporting bacterial growth and maintaining a stable microbial community. Nucleotide biosynthesis (purine and pyrimidine *de novo* biosynthesis) is critical in DNA replication, energy storage, and intracellular signaling (Goncheva et al., 2022). Finaly, tetrapyrrole biosynthesis is involved in heme production, a key molecule that influences bacterial survival and interactions with host systems (Stasiuk et al., 2021).

### 4.4 Limitations and strengths of the study

A major limitation of most studies investigating the human oral microbiome, including ours, is the small sample size, often constrained by the high costs associated with sequencing methods, particularly shotgun metagenomic sequencing. Given the highly individualized nature of the oral microbiome, shaped by factors such as age, gender, diet, lifestyle, genetic predispositions, and the presence of systemic diseases, future research should prioritize larger and more diverse cohorts spanning various regions and countries to enhance the generalizability and applicability of the findings.

Further longitudinal studies are needed to explore changes in the oral microbiome during the transition from health to disease to advance precision and personalized medicine (Belibasakis et al., 2019). The development of tools for individualized microbiome profiling (personalized metagenomics) holds great potential for applications in microbiome-based medicine (Kim et al., 2024).

Our findings indicate that healthy implants in an oral cavity without any PI-affected implants show distinct microbial signatures compared to healthy implants co-occurring with PI-affected implants in the same oral cavity. The bacterial species and functional pathways associated with healthy implants co-occurring with PI-affected implants more closely resemble those related to PI than those associated with healthy implants in PI-free oral cavities. This suggests that the microbiome and functional profile of implants diagnosed as healthy differ depending on their context within the oral cavity. Also, these microbial and functional biomarkers follow the same pattern in salivary samples. PI-affected implants may serve as a reservoir for a different microbial niche, influencing changes in the microbiome of healthy implants and saliva. These findings also highlight saliva’s potential as a convenient and non-invasive medium for identifying biomarkers related to PI diagnosis and prevention. With ongoing advancements in metagenomics and studies like ours, there is a growing opportunity to identify biomarkers that can be validated and translated into clinical applications. This has the potential to greatly enhance early diagnosis, treatment monitoring, and personalized management of PI. Such advancements are of significant clinical importance, paving the way for potential breakthroughs in understanding and managing PI, with important implications for both clinicians and researchers.

## Data Availability

The datasets presented in this study can be found in a online repository, NCBI SRA database, under accession number PRJNA1163384 (https://www.ncbi.nlm.nih.gov/sra/?term=PRJNA1163384).
