# Supplementary material for: Linking peri-implantitis to microbiome changes in affected implants, healthy implants, and saliva: a cross-sectional pilot study
Source: Front Cell Infect Microbiol. 2025 Apr 17;15:1543100. doi: 10.3389/fcimb.2025.1543100 (PMC12043654; doi:10.3389/fcimb.2025.1543100)
Supplement: Supplementary file 1 [file DataSheet1.zip › Supplementary Materials/Supplementary_Material.docx]

Supplementary Material

## Supplementary Figures


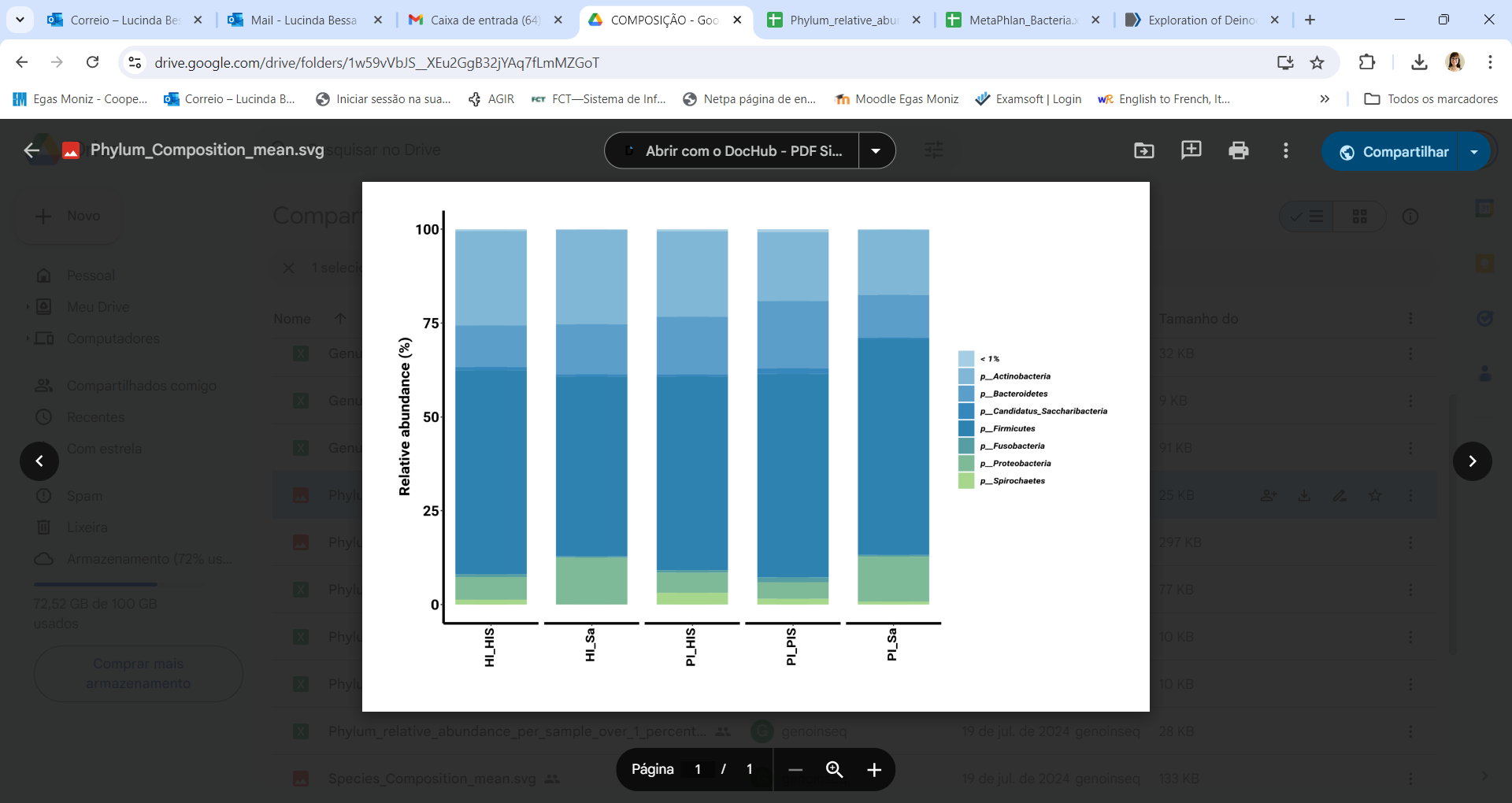


**Supplementary Figure 1.** Relative abundances (>1%) of the bacterial composition at the phylum level in each study group.


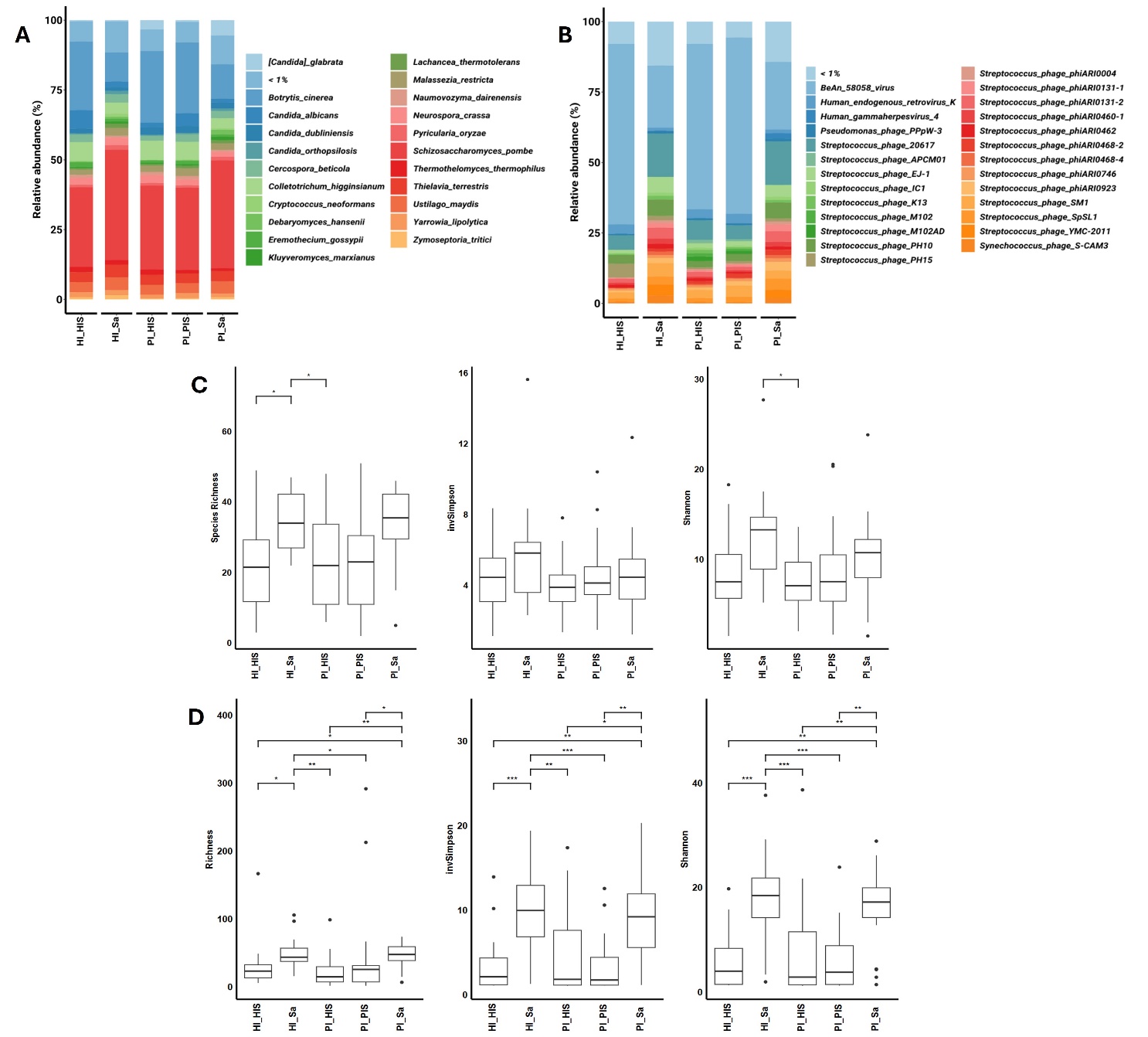


**Supplementary Figure 2.** Relative abundances (>1%) of the fungi (**A**) and virus (**B**) composition at the species level in each study group. ) Hill diversity indices of the fungi (**C**) and virus (**D**) communities of the five study groups. * p < 0.05, ** p < 0.01, *** p < 0.001, based on the Kruskal-Wallis rank.


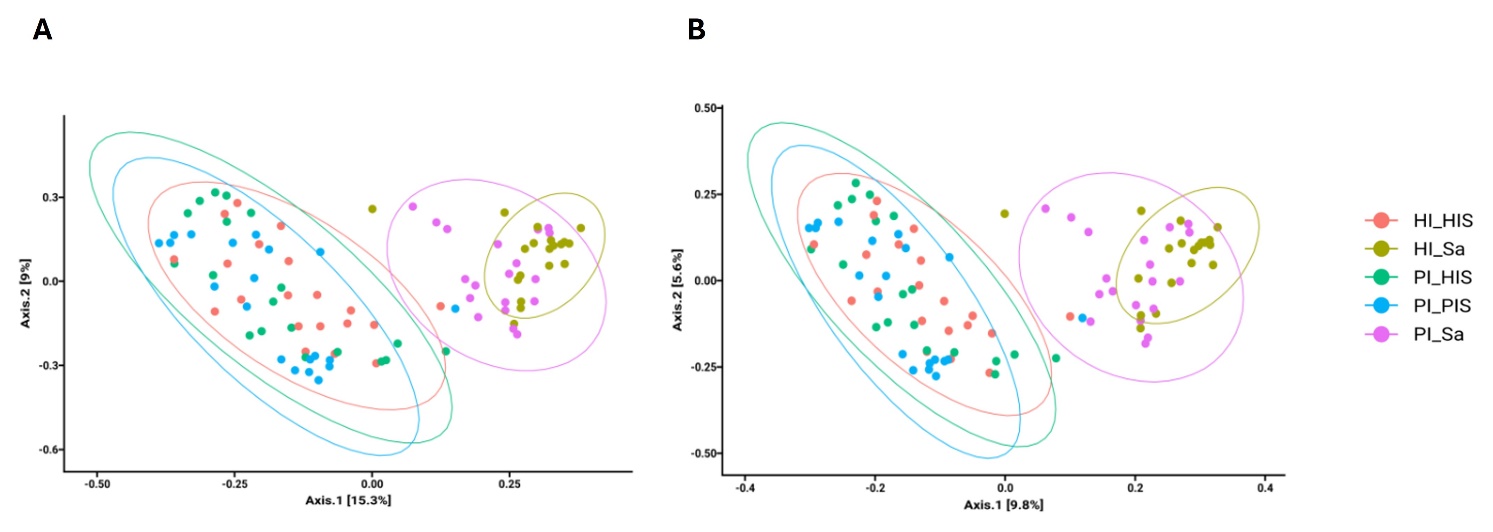


**Supplementary Figure 3.** Relative PCoA of Bray-Curtis distance (**A**) and Jaccard (**B**) depicting the beta diversity between the groups. pairwise PERMANOVA: differences between any two of the three groups (HI_HIS, PI_HIS and PI_PIS) and between HI_Sa and PI_Sa were not statistically significant (p > 0.1). Differences, were, however, significant between saliva and subgingival biofilm groups (p < 0.001).


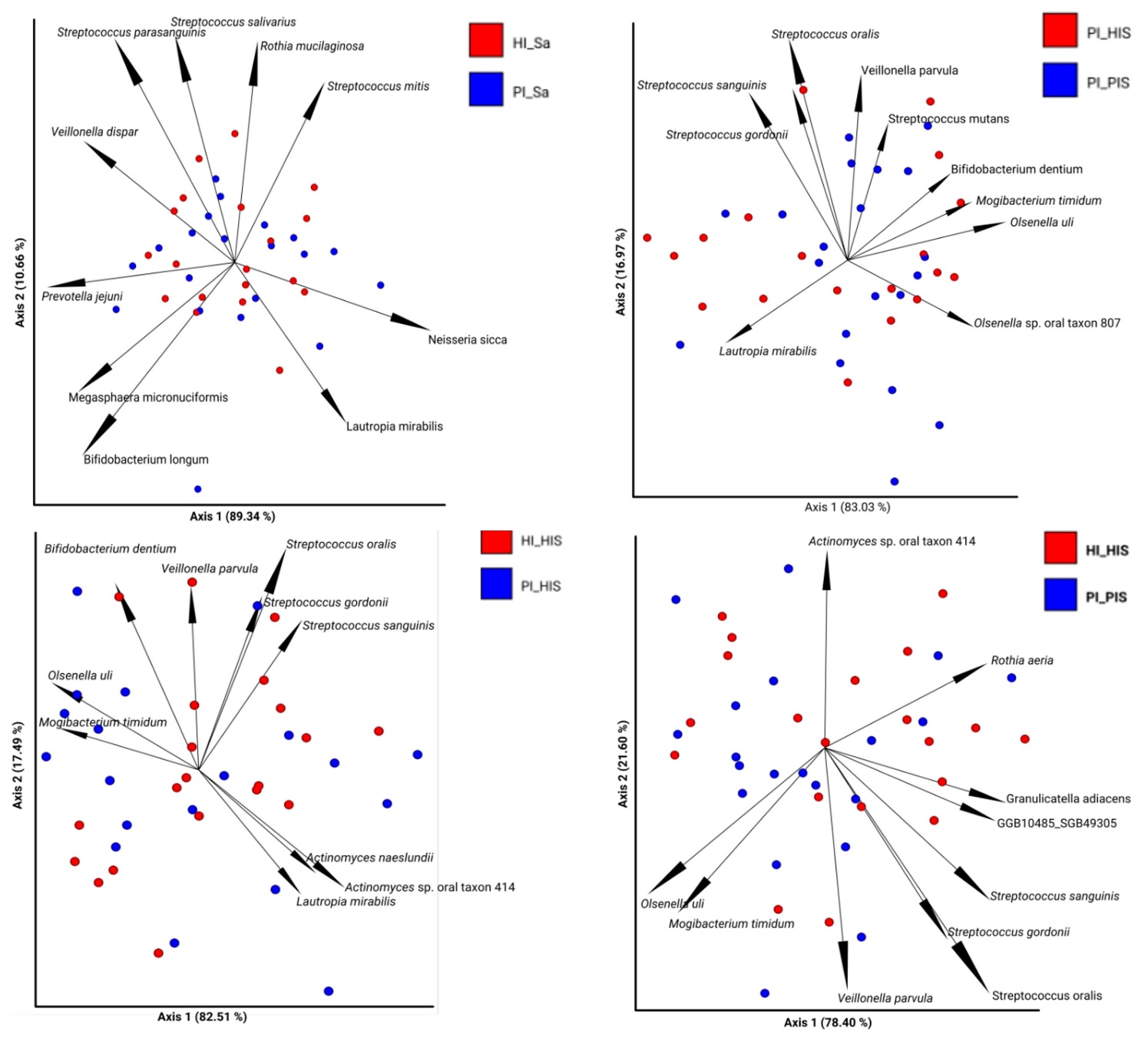


**Supplementary Figure 4.** Beta diversity biplots generated using DEICODE robust Aitchison PCA. Data points represent individual samples. The arrows illustrate feature loadings (i.e., bacterial species that drive differences in the ordination space). PERMANOVA analysis of beta diversity revealed no significant differences (p > 0.05) between the groups regarding their bacterial composition.


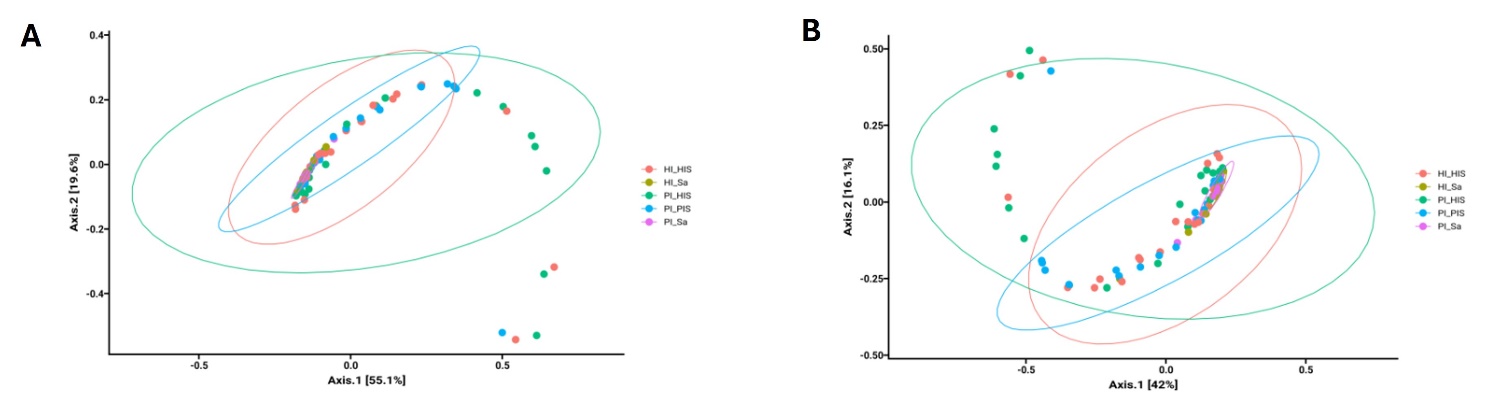


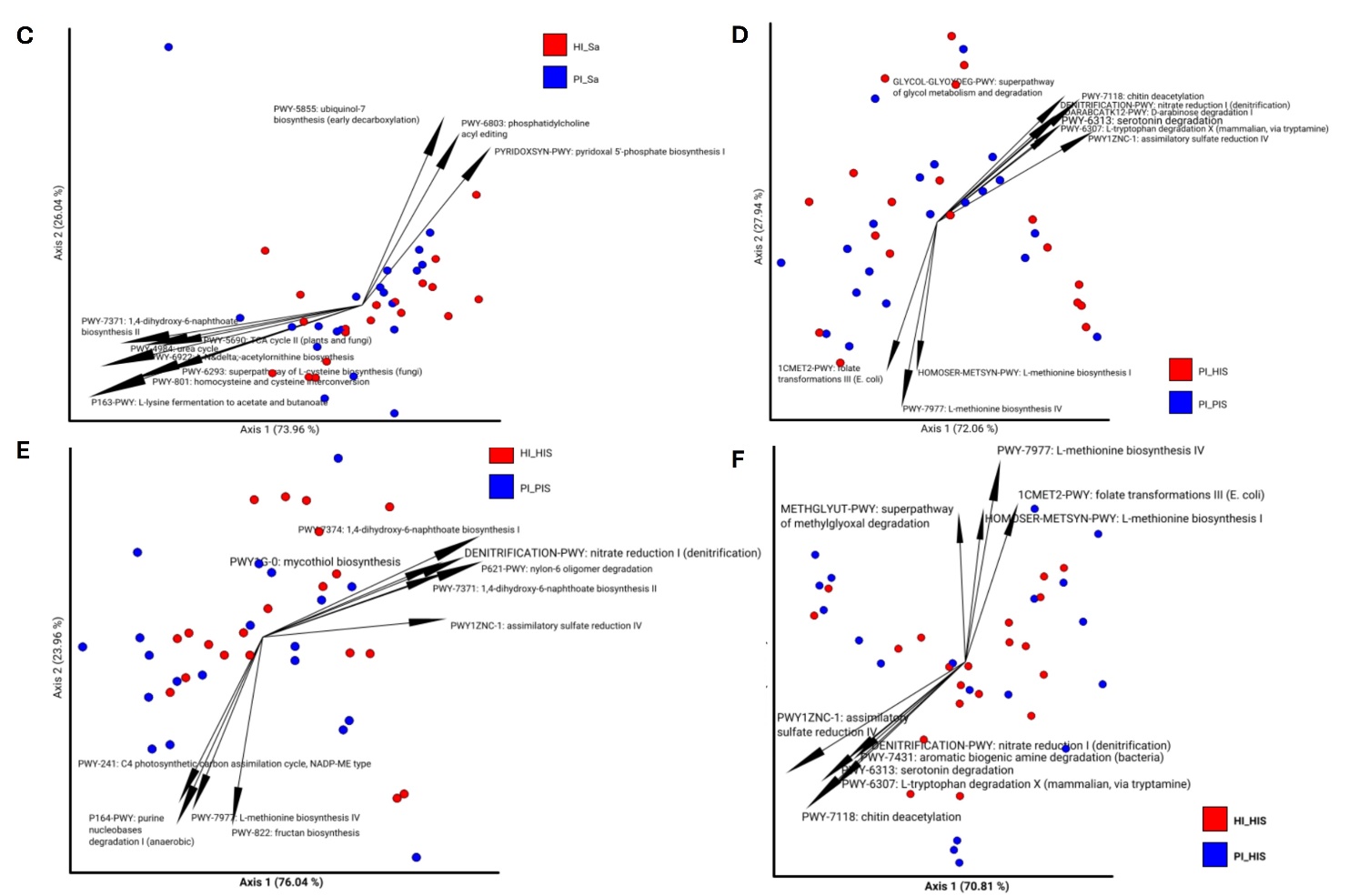


**Supplementary Figure 5.** Relative PCoA of Bray-Curtis distance (**A**) and Jaccard (**B**), and beta diversity biplots generated using DEICODE robust Aitchison PCA (**C**, **D**, **E** and **F**) depicting the beta diversity of functional pathways between the groups. pairwise PERMANOVA: differences between any two of the three groups (HI_HIS, PI_HIS and PI_PIS) and between HI_Sa and PI_Sa were not statistically significant (p > 0.05). Differences, were, however, significant between saliva and subgingival biofilm groups (p < 0.01).

## Supplementary Tables

**Supplementary Table 1.** PERMANOVA e PAIRWISE PERMANOVA results of bacteria (**A**), fungi (**B**) and viruses (**C**) for the study groups.

| 1. **Bacteria** |  | |  | |  | |  | |  | |  | |
| --- | --- | --- | --- | --- | --- | --- | --- | --- | --- | --- | --- | --- |
| **PERMANOVA results** |  | |  | |  | |  | |  | |  | |
| method name | PERMANOVA | |  | |  | |  | |  | |  | |
| test statistic name | pseudo-F | |  | |  | |  | |  | |  | |
| sample size | 100 | |  | |  | |  | |  | |  | |
| number of groups | 5 | |  | |  | |  | |  | |  | |
| test statistic | 37,303046 | |  | |  | |  | |  | |  | |
| p-value | 0,001 | |  | |  | |  | |  | |  | |
| number of permutations | 999 | |  | |  | |  | |  | |  | |
| **PAIRWISE PERMANOVA** |  | |  | |  | |  | |  | |  | |
| **Group 1** | **Group 2** | | **Sample size** | | **Permutations** | | **pseudo-F** | | **p-value** | | **q-value** | |
| HI_HIS | HI_Sa | | 40 | | 999 | | 87,501549 | | 0,001 | | 0,001667 | |
|  | PI_HIS | | 40 | | 999 | | 0,509927 | | 0,571 | | 0,571 | |
|  | PI_PIS | | 40 | | 999 | | 0,73118 | | 0,489 | | 0,543333 | |
|  | PI_Sa | | 40 | | 999 | | 48,725857 | | 0,001 | | 0,001667 | |
| HI_Sa | PI_HIS | | 40 | | 999 | | 59,94133 | | 0,001 | | 0,001667 | |
|  | PI_PIS | | 40 | | 999 | | 112,004872 | | 0,001 | | 0,001667 | |
|  | PI_Sa | | 40 | | 999 | | 2,859605 | | 0,074 | | 0,105714 | |
| PI_HIS | PI_PIS | | 40 | | 999 | | 2,114631 | | 0,124 | | 0,155 | |
|  | PI_Sa | | 40 | | 999 | | 33,168077 | | 0,001 | | 0,001667 | |
| PI_PIS | PI_Sa | | 40 | | 999 | | 64,024418 | | 0,001 | | 0,001667 | |
|  |  | |  | |  | |  | |  | |  | |
| 1. **Fungi** | | |  |  |  |  |  |  |  |  |  |  |
| **PERMANOVA results** |  | |  | |  | |  | |  | |  | |
| method name | PERMANOVA | |  | |  | |  | |  | |  | |
| test statistic name | pseudo-F | |  | |  | |  | |  | |  | |
| sample size | 100 | |  | |  | |  | |  | |  | |
| number of groups | 5 | |  | |  | |  | |  | |  | |
| test statistic | 0,893837 | |  | |  | |  | |  | |  | |
| p-value | 0,522 | |  | |  | |  | |  | |  | |
| number of permutations | 999 | |  | |  | |  | |  | |  | |
|  |  | |  | |  | |  | |  | |  | |
| **PAIRWISE PERMANOVA** |  | |  | |  | |  | |  | |  | |
| **Group 1** | **Group 2** | | **Sample size** | | **Permutations** | | **pseudo-F** | | **p-value** | | **q-value** | |
| HI_HIS | HI_Sa | | 40 | | 999 | | 2,14657313 | | 0,129 | | 0,506667 | |
| HI_HIS | PI_HIS | | 40 | | 999 | | 0,04323615 | | 0,947 | | 0,947 | |
| HI_HIS | PI_PIS | | 40 | | 999 | | 0,10258629 | | 0,867 | | 0,947 | |
| HI_HIS | PI_Sa | | 40 | | 999 | | 0,98428639 | | 0,346 | | 0,688571 | |
| HI_Sa | PI_HIS | | 40 | | 999 | | 2,14596313 | | 0,152 | | 0,506667 | |
| HI_Sa | PI_PIS | | 40 | | 999 | | 1,92593567 | | 0,149 | | 0,506667 | |
| HI_Sa | PI_Sa | | 40 | | 999 | | 0,80618615 | | 0,479 | | 0,688571 | |
| PI_HIS | PI_PIS | | 40 | | 999 | | 0,15934407 | | 0,798 | | 0,947 | |
| PI_HIS | PI_Sa | | 40 | | 999 | | 1,15847092 | | 0,27 | | 0,675 | |
| PI_PIS | PI_Sa | | 40 | | 999 | | 0,62495122 | | 0,482 | | 0,688571 | |
|  |  | |  | |  | |  | |  | |  | |
| 1. **Viruses** |  | |  | |  | |  | |  | |  | |
| **PERMANOVA results** |  |  | |  | |  | |  | |  | |  |
| method name | PERMANOVA |  | |  | |  | |  | |  | |  |
| test statistic name | pseudo-F |  | |  | |  | |  | |  | |  |
| sample size | 100 |  | |  | |  | |  | |  | |  |
| number of groups | 5 |  | |  | |  | |  | |  | |  |
| test statistic | 8,393636 |  | |  | |  | |  | |  | |  |
| p-value | 0,001 |  | |  | |  | |  | |  | |  |
| number of permutations | 999 |  | |  | |  | |  | |  | |  |
|  |  |  | |  | |  | |  | |  | |  |
| **PAIRWISE PERMANOVA** |  |  | |  | |  | |  | |  | |  |
| **Group 1** | **Group 2** | **Sample size** | | **Permutations** | | **pseudo-F** | | **p-value** | | **q-value** | |  |
| HI_HIS | HI_Sa | 40 | | 999 | | 23,3154174 | | 0,001 | | 0,005 | |  |
| HI_HIS | PI_HIS | 40 | | 999 | | 1,54570163 | | 0,193 | | 0,275714 | |  |
| HI_HIS | PI_PIS | 40 | | 999 | | 1,1374078 | | 0,314 | | 0,3925 | |  |
| HI_HIS | PI_Sa | 40 | | 999 | | 21,9933345 | | 0,001 | | 0,005 | |  |
| HI_Sa | PI_HIS | 40 | | 999 | | 9,66832793 | | 0,004 | | 0,008 | |  |
| HI_Sa | PI_PIS | 40 | | 999 | | 12,1786044 | | 0,002 | | 0,005 | |  |
| HI_Sa | PI_Sa | 40 | | 999 | | 0,02557967 | | 0,967 | | 0,967 | |  |
| PI_HIS | PI_PIS | 40 | | 999 | | 0,20483098 | | 0,798 | | 0,886667 | |  |
| PI_HIS | PI_Sa | 40 | | 999 | | 9,01642057 | | 0,005 | | 0,008333 | |  |
| PI_PIS | PI_Sa | 40 | | 999 | | 11,2956526 | | 0,002 | | 0,005 | |  |
|  |  |  | |  | |  | |  | |  | |  |
